# Toll-like receptors and toll-like receptor-targeted immunotherapy against glioma

**DOI:** 10.1186/s13045-021-01191-2

**Published:** 2021-10-29

**Authors:** Yang Xun, Hua Yang, Bozena Kaminska, Hua You

**Affiliations:** 1 Department of Basic Medicine and Biomedical Engineering, School of Medicine, Foshan University, Foshan, 528000 Guangdong Province China; 2 Affiliated Cancer Hospital and Institute of Guangzhou Medical University, No.78 Heng-Zhi-Gang Road, Yue Xiu District, Guangzhou, 510095 China; 3 Laboratory of Molecular Neurobiology, Nencki Institute of Experimental Biology, Warsaw, Poland

**Keywords:** Glioma, Toll-like receptors, TLR-targeted therapies, Clinical trials, Immunotherapy

## Abstract

Glioma represents a fast proliferating and highly invasive brain tumor which is resistant to current therapies and invariably recurs. Despite some advancements in anti-glioma therapies, patients’ prognosis remains poor. Toll-like receptors (TLRs) act as the first line of defense in the immune system being the detectors of those associated with bacteria, viruses, and danger signals. In the glioma microenvironment, TLRs are expressed on both immune and tumor cells, playing dual roles eliciting antitumoral (innate and adaptive immunity) and protumoral (cell proliferation, migration, invasion, and glioma stem cell maintenance) responses. Up to date, several TLR-targeting therapies have been developed aiming at glioma bulk and stem cells, infiltrating immune cells, the immune checkpoint axis, among others. While some TLR agonists exhibited survival benefit in clinical trials, it attracts more attention when they are involved in combinatorial treatment with radiation, chemotherapy, immune vaccination, and immune checkpoint inhibition in glioma treatment. TLR agonists can be used as immune modulators to enhance the efficacy of other treatment, to avoid dose accumulation, and what brings more interests is that they can potentiate immune checkpoint delayed resistance to PD-1/PD-L1 blockade by upregulating PD-1/PD-L1 overexpression, thus unleash powerful antitumor responses when combined with immune checkpoint inhibitors. Herein, we focus on recent developments and clinical trials exploring TLR-based treatment to provide a picture of the relationship between TLR and glioma and their implications for immunotherapy.

## Introduction

### Gliomas

Gliomas are the most common primary malignant tumors of the central nervous system (CNS) accounting for 30% of all primary brain tumors, and 80% of malignant ones, and are responsible for the majority of death from primary brain tumors [1]. Gliomas in adults include astrocytoma, anaplastic astrocytoma, oligodendrogliomas, anaplastic oligodendrogliomas, glioblastoma (GBM), and less common gliomas such as pilocytic astrocytoma, pleomorphic xanthoastrocytoma, and ependymoma [2]. In children, the most common types of gliomas are pilocytic astrocytoma and diffuse intrinsic poutine gliomas of various grades [3]. Based on histological features, gliomas are classified into World Health Organization (WHO) grades I–IV: grades I and II are considered low-grade gliomas (LGG), and grades III and IV are considered high-grade gliomas (HGG). GBM is a grade IV glioma which accounts for 60% of all gliomas and has the worst survival [2].

### Standard treatment for glioma

Standard treatments for HGG include maximal surgical resection, radiotherapy, and temozolomide (TMZ) administration. However, due to incomplete surgical resection, the immunosuppressive tumor microenvironment (TME), and the presence of the blood–brain barrier hampering the transport of chemotherapeutic agents, the overall prognosis of malignant glioma remains poor [4]. Based on the clinical practice guidelines for the management of adult diffuse glioma published by Chinese Glioma Cooperative Group (CGCG), China was one of the top countries with the largest incident cases (56 per 100,000 individuals worldwide) and the most deaths of glioma [5]. The median overall survival (OS) times were 78.1, 37.6, and 14.4 months for LGG, anaplastic gliomas, and GBM, respectively [5]. In the situation of GBM relapse, there is no standard therapy and the OS is less than 9 months [6]. Although GBM is not so common in pediatric patients, once found the OS is only 10 months with the current treatment [7].

### Molecular markers of glioma

The absence or presence of certain molecular genetic features is used as indicators of prognosis in patients. For example, mutation in the isocitrate dehydrogenase (*IDH*) 1 or 2 gene is commonly found in human LGG, grade III glioma, and secondary GBM and has been used as a new decisive marker for glioma classification since 2016 [2]. Astrocytoma or anaplastic astrocytoma are grouped into *IDH*-mutant, *IDH* wild type, and not otherwise specified (NOS) categories. Oligodendroglioma or anaplastic oligodendrogliomas are mainly featured by *IDH* mutation, chromosomal 1p/19q codeletion, and O-6-methyguannine-DNA methyltransferase (*MGMT*) promoter methylation, which confer a favorable prognosis [5, 8]. IDH wild-type GBM account for 90% of all GBM cases and is an indicator of poor prognosis. Other genomic alterations of GBM include telomerase reverse transcriptase (*TERT*) promoter mutation, epidermal growth factor receptor (*EGFR*) amplification, and tumor suppressor phosphatase and tensin homolog on chromosome 10 (*PTEN*) loss/mutation. *IDH*-mutant GBM is considered less aggressive with *TP53* and *ATRX* mutations [2]. Grade II and III *IDH* wild-type glioma in adults behave equivalent to GBM, especially when they have *TERT* promoter mutation, *EGFR* amplification, and/or chromosome 7 gain and chromosome 10 loss [2]. In comparison, *IDH* wild-type gliomas in pediatric or young adult patients are genetically featured with BRAF^V600E^ mutation, fibroblast growth factor receptor 1 (*FGFR1*) alteration, and a MYB or MYB1 rearrangement [9].

### Targeted treatment for glioma

In recent years, growing knowledge of tumor genomics has led to novel therapies against glioma. Given *IDH1/2* mutation is common in LGG, direct targeting of the mutant enzyme has proven to be promising in preclinical models. Ivosidenib (AG-120) has shown positive results in *IDH*-mutant advanced glioma [10]; other *IDH* mutation inhibitors, such as AGI-5198 [11], BAY1436032 [12], vorasidenib (AG-881) [13], enasidenib (AG-221) [14], and DS-1001b [15], are still in early clinical development, and their efficacy and toxicity need subsequent studies.

Multiple signaling pathways or genes are dysregulated in glioma cells, including PI3K/mTOR, retinoblastoma, epidermal growth factor (EGF), TP53, and vascular endothelial growth factor (VEGF). Signaling inhibitors, enzyme inhibitors, and receptor antibodies have been designed to target altered signaling pathways and genes against glioma. The PI3K/mTOR is one of the most altered molecular pathways in *IDH* wild-type glioma, as a consequence of gene function loss such as tumor suppressor *PTEN*. The use of common PI3K pathway inhibitors (*e.g.*, buparlisib and temsirolimus) has turned out to be challenging in clinical application; insufficient inhibition and unfavorable tolerability were often observed [16]. Other PI3K pathway inhibitor (everolimus) also showed limited roles in clinical trials against GBM [17]. The retinoblastoma pathway is altered in the majority of *IDH* wild-type GBM. Drugs that are designed to inhibit retinoblastoma pathway either showed disappointing (palbociclib) or yet-to-be-confirmed (TG02) results [18]. Targeting EGFR in *IDH* wild-type HGG with tyrosine kinase inhibitors has been explored. EGFR class III variant is a constitutively active form of EGFR that is commonly expressed in GBM. Clinical trials showed survival signal in EGFRvIII^+^ recurrent GBM, but not in newly diagnosed GBM [19]. Tumor-specific antibody drug conjugate Depatux-M which consists of an EGFR antibody (ABT-806) linked to monomethyl auristatin F provided positive results in recurrent EGFR-amplified GBM, but not in phase III trial due to unstable EGFR expression [20]. *TP53* tumor suppressor gene is among the most studied genes in HGG. Restoring the function of its main gene product p53 has been studied but only showed limited success [21]. Antiangiogenic antibody regorafenib targeting VEGF was proven to increase survival in patients with recurrent GBM [22].

Other potential targets, such as *TERT* promotor mutation, although is considered to be the most common molecular alterations in *IDH* wild-type GBM, have not become a major target for glioma therapy yet [23]. *MGMT* promotor methylation has been established as a predictive biomarker for clinical benefit only in newly diagnosed glioma. FGFR and BRAF^V600E^ mutations are considered as potential drug targets for intervention, but their relevance is limited to patients with tumors exhibiting FGFR-TACC fusions [24] and BRAF^V600E^-mutant GBM [25].

### Immunotherapy for glioma

Up to date, different immunotherapies have been explored in treating glioma, especially in HGG patients. In glioma, high PD-1/PD-L1 expression in tumor cells has been correlated with poor patient prognosis through immune suppression; this has attracted great interests in antibody development preventing the association between programmed cell death protein 1 (PD-1)/PD-ligand 1 (PD-L1) [26]. To date, anti-PD-1 and anti-PD-L1 antibodies, although have achieved promising results against various solid tumors, have not shown marked success in most GBM cases (such as CheckMate-143, CheckMate-498, CheckMate-548, and nivolumab alone) [27, 28]. Combination of anti-PD-L1 treatment with cytotoxic T-lymphocyte associated protein 4 blockade, which reactivates T-cell functions, is currently under phase III trial in recurrent GBM patients (NCT02017717).

Alternative immunotherapy includes chimeric antigen receptor (CAR) T-cell therapy which is a challenging strategy in terms of identification of tumor-specific or tumor-associated antigens. Positive results using IL13Rα2-CAR-T on recurrent multifocal GBM patient have been reported without severe toxic effect in some clinical trials [29]. At present, a number of CAR-T-cell therapy targets have been reported in glioma treatment, such as EGFRvIII-CAR-T [30], CSPG4-CAR-T [31], HER2 [32], EphA2 [33], and B7H3 [34].

Vaccination appears to be a feasible approach by eliciting antitumor immune response through antigen presentation to T-cells. Peptide vaccines rindopepimut targeting EGFRvIII showed improved results when combined with bevacizumab in relapsed EGFRvIII^+^ GBM [35], but failed in newly diagnosed GBM when combined with TMZ [19] due to unstable EGFRvIII expression. Tumor lysate-pulsed dendritic cell (DC) vaccine (DCVax®-L) [36] and Glioma Actively Personalized Vaccine Consortium (GAPVAC) [37] resulted in longer OS and progression-free survival (PFS) in GBM patients. However, vaccination is a complicated and personalized approach that requires intensive testing to evaluate translation into clinical benefit as monotherapy [27].

Oncolytic viruses constitute a potential therapeutic approach in GBM by activating immune system through pathogen-associated molecular patterns (PAMPs), pattern recognition receptors, and macrophages. Some oncolytic viral therapies (recombinant non-pathogenic polio–rhinovirus chimera [38] and replication-deficient adenoviruses [39]) showed favorable prognosis in phase II clinical trials on recurrent GBM patients but not in phase III trials.

During tumor progression, glioma cells interact with the surrounding microenvironment composed of astrocytes, endothelial cells, and numerous residing and infiltrating immune cells to produce cytokines, chemokines, and extracellular proteins, which in turn promotes immune evasion and supports tumorigenesis [40, 41]. Although the results of current targeted and immune therapies are sometimes disappointing for HGG, combination approaches or reversing the immunosuppressing microenvironment attract interests.

### Toll-like receptors as potential therapeutic targets in glioma

**Fig. 1:**
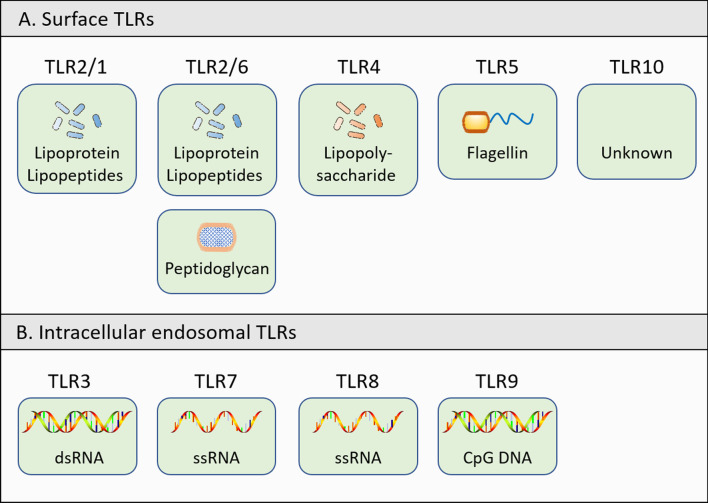
Human TLRs and their corresponding PAMPs. TLR1, 2, 4, 5, 6, and 10 are anchored on the cell membranes and are in charge of defensing against extracellular pathogens; TLR3, 7, 8, and 9 are located in the endosome to detect intracellular pathogens. TLRs bind to their corresponding ligands to induce physiological and/or pathological downstream signaling

Toll-like receptors (TLRs) are ubiquitously expressed pathogen recognition receptors. They act as the first line of defense in the innate immune system and become activated upon detection of PAMPs or products released during cell breakdown, known as danger-associated molecular patterns (DAMPs) [42]. The human TLR family consists of 10 receptors (TLR1−10) grouped into two major categories: surface TLRs (TLR1, TLR2, TLR4, TLR5, TLR6, and TLR10), which reside on the plasma membrane and bind to microbial-derived ligands, and intracellular endosomal TLRs (TLR3, TLR7, TLR8, and TLR9), which are nucleic acid-sensing TLRs, responding to DNA/RNA derived from pathogens and dead cells (Fig. 1) (Table 1) [43].

**Table 1:** The structures and sizes of human TLRs

Name	Alias	Location	No. of amino acids	Protein size (kDa)	Structure code
TLR1	CD281, TIL, TIL, LPRS5, rsc786	4p14	786	90.18	6NIH [44]
TLR2	CD282, TIL4	4q31.3	784	89.85	6NIG [44]
TLR3	CD283, IIAE2	4q35.1	904	103.84	2A0Z [45]
TLR4	ARMD10, CD284, TLR-4, TOLL	9q33.1	839	95.7	3FXI [46]
TLR5	MELIOS, SLE1, SLEB1, TIL3	1q41	858	97.82	3J0A [47]
TLR6	CD286	4p14	796	91.9	3A79 [48]
TLR7	IMD74, TLR7-like	Xp22.2	1049	120.93	5GMH [49]
TLR8	CD288	Xp22.2	1059	121.78	6KYA [50]
TLR9	CD289	3p21.2	1032	115.88	None
TLR10	CD290	4p14	811	94.58	None

**Fig. 2:**
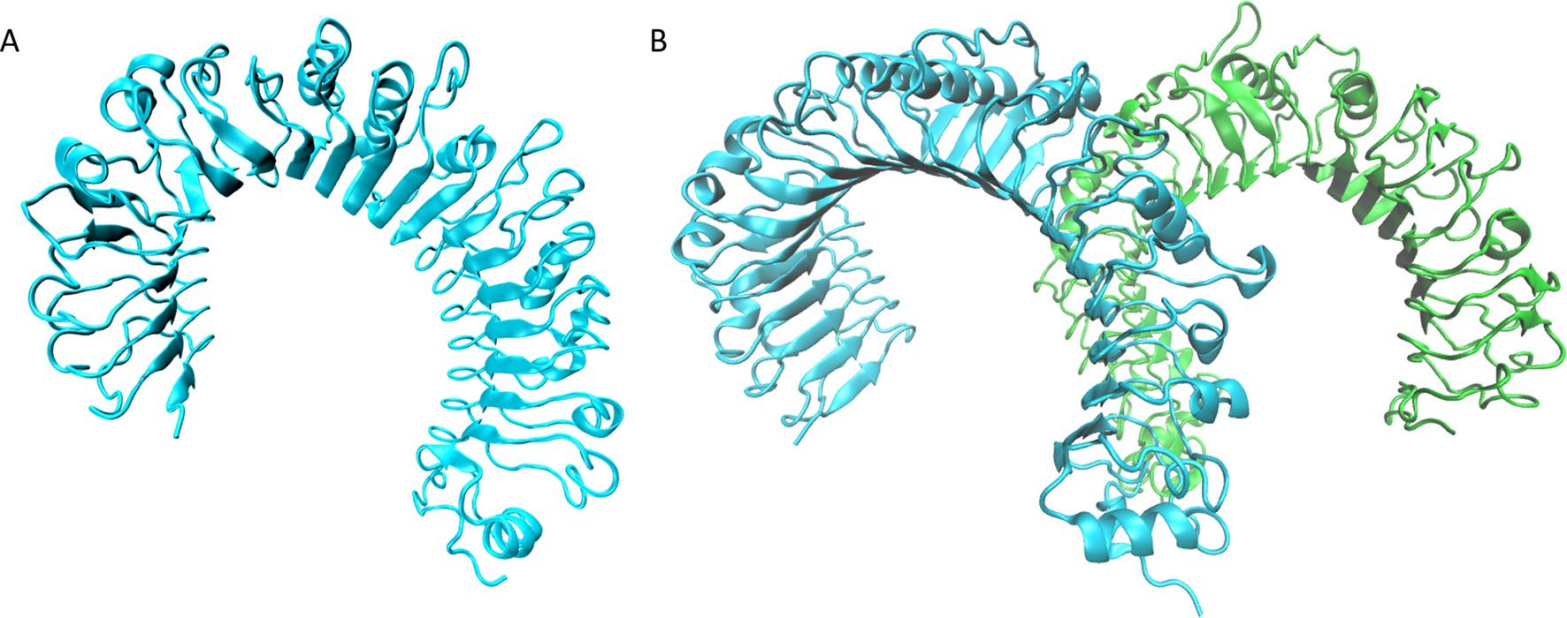
Overall structures of TLR monomer and dimer. **a** and **b** represents TLR1 monomer (PDB#6NIH) and TLR1 (cyan)-TLR2 (green) dimer complex induced by binding to ligand (PDB#6NIG), respectively. The structures were reconstructed using the VMD software

All the extracellular domains of TLRs possess 16–28 hydrophobic leucine-rich repeat (LRR) modules sandwiched between the C- and N-terminus. The presence of LRR modules led to a common horseshoe-shaped structures in all TLRs, exposing the hydrophilic area to the solvent. Each individual LRR module is 20–30 amino acids long and includes a conserved ‘LxxLxxLxxN’ motif that forms parallel β-sheets and a variable region that exhibits divergence as a result of exposure to diversified PAMPs [51]. The structures of TLR1, 2, and 4 revealed two structural transitions in the central β-sheet, dividing the LRR domains into N-terminal, C-terminal, and a central subdomain which vary considerably in size, contributing to the ligand-binding functions of TLRs [52, 53]. In contrast, TLR3 has flat horseshoe-like shape and the size of LRR modules is less variable [54]. Sequence analysis of TLR5, 7, 8, and 9 demonstrated that they belong to the single-domain subfamily with relatively unvarying module length, while TLR6 and 10 exhibit distorted central subdomain conformation similar to TLR1, 2, and 4 [51]. Binding of ligands induces ‘M’-shaped TLR dimerization, which then triggers the recruitment of adaptor proteins to the intracellular Toll/IL-1R (TIR) domains of TLRs for initiation of various downstream signaling [52] (Fig. 2).

Over the past decade, TLRs have been reported to trigger immune responses in various tumor types [55–57]. In the CNS, TLRs are expressed in neurons, glial cells, and immune cells. There is evidence that TLRs play important roles both in cancer cells and in the modulation of immune responses in glioma. Upon ligand recognition, TLRs activate intracellular cascades to promote downstream signaling, supporting either tumor progression or suppression [58], and therefore can be used as potential targets in cancer therapy. This review reports TLR expression and mechanisms of action in glioma and focuses on the development of TLR-based immunotherapies against gliomas.

## TLR expressions in gliomas

### TLR expressions in glioma cells

The expression of all ten types of TLRs has been found in human brain. In glioma, elevated TLR1, TLR2, TLR4, TLR5, TLR6, and TLR9 expressions were observed in tumor cell lines and tissues compared with non-neoplastic brain tissues, particularly in the mesenchymal subtype of GBM [55, 59, 60]. TLR2 recognizes gram-positive bacterial molecules (lipopeptides, lipoteichoic acids, or peptidoglycan) in association with TLR1 or TLR6 and plays an important role in the immune system that is expressed in both immune cells and cancer cells. *TLR2* mRNA and protein levels in glioma tissues were found positively associated with glioma WHO histological grades and poor clinical outcome, and its overexpression could enhance glioma cell activity and cell cycle progression [61, 62]. *TLR4* mRNA and protein expression have been detected in U118, U87, A172, and LN229 glioma cell lines [63]. In tissues and primary biopsies from glioma patients, the expression of TLR4 was significantly higher in grade IV GBM than in grade III anaplastic astrocytoma, correlating with poor prognosis [55, 64, 65]. Upon recognition of endogenous ligands derived from dead cells and lipopolysaccharides (LPSs), TLR4-mediated signaling was shown to associate with the regulation of cell survival, proliferation, migration, immune evasion, and resistance to tumor necrosis factor α (TNF-α) treatment [64]. Diminishing TLR4 expression abrogated GBM invasiveness, upregulated apoptosis, and impaired survival signaling [66]. TLR9 was reported to express on human glioma cell line U251, U87, primary human glioma biopsies, and isolated human glioma stem cells (GSCs). Increased expression of TLR9 was associated with higher glioma grade and worse prognosis [59, 60, 67, 68]. In supratentorial GBM cases, patients with low TLR9 expression tend to have longer survival than those with low TLR9 expression [60]. Up to date, TLR7/8 expression was found absent in glioma cell lines (CNS-1 and GL-261) [69], and TLR10 has unknown ligand and was poorly investigated. However, the analysis of The Cancer Genome Atlas (TCGA) database showed that high *TLR10* expression in GBM patients may associate with tumor grade, poor OS, and PFS [70].

### TLR expressions in glioma microenvironment

#### TLR expressions on microglia

The brain tumor site contains various cell types, including tumor cells and immune cells. Microglia are CNS-resident macrophages that play crucial roles in initiating local inflammation. Microglia of human brain express a wide profile of TLRs (TLR1–9) (Table 2), regulating various secreted inflammatory mediators. The expression of microglial TLRs increases with the exposure to pathogen attack or other proinflammatory stimuli, responsible for brain’s innate immune system [71].

**Table 2:** Summary of expression of TLR in CNS cells and tissues

TLRs	Species	Tissue(s)/cell type(s)	Expression	Anti-/pro-tumorigenic	References
TLR1	Human	Glioma tissues	Upregulated	Pro-	[55, 61, 62]
		Glioma cell lines (U87-MG, A172)	Upregulated	Pro-	[61, 62]
		Microglia	Upregulated	Anti-	[71, 72]
		GAMs	Upregulated	Pro-	[62, 75–78]
	Mouse	Microglia	Upregulated	Anti-	[71, 72]
		GAMs	Upregulated	Pro-	[62, 75–78]
TLR2	Human	Glioma tissue	Upregulated	Pro-	[55, 61, 62]
		Glioma cell lines (U87-MG, A172, GL261)	Upregulated	Pro-	[55, 61, 62, 88]
		Microglia	Upregulated	Anti-	[71, 72]
		GAMs	Upregulated	Pro-	[62, 75–78]
		mDCs	Upregulated	Anti-	[83, 84]
		GSCs	Upregulated	Pro-	[88] [86]
	Mouse	Microglia	Upregulated	Anti-	[71, 72]
		GAM	Upregulated	Pro-	[62, 75–78]
		GSCs of murine GL261 cell line	Present Present	n.s n.s	[86, 88]
TLR3	Human	Glioma tissues	Upregulated	n.s	[62, 78]
		GBM (U87-MG, A172, U251, LN229)	Upregulated	n.s	[55, 95, 96]
		Microglia	Upregulated	Anti-	[71, 79]
	Mouse	GBM (GL261)	Upregulated	n.s	[95, 96]
		Microglia	Present	n.s	[71, 79]
TLR4	Human	Glioma tissue	Upregulated	Pro-	[55]
		Glioma cell lines (U87-MG, A172, LN229, U118, SF126, U87, U251, GI261)	Upregulated	Pro-	
		GAMs	Upregulated	Pro-tumor	[93, 94]
		GSCs	Upregulated	Pro/anti-	[86, 88, 90–92]
	Mouse	Glioma cell lines (GL261)	Upregulated	Pro-	[95, 97, 98]
		GSC	Upregulated	Pro/anti-	[90, 99, 100]
TLR5	Human	Glioma tissues	Upregulated	n.s	[55]
		GBM (U87-MG, A172)	Upregulated	n.s	[55, 72, 73]
		Microglia	Upregulated	Anti-	[71, 72]
	Mouse	GBM (G261, T98G)	Upregulated	n.s	[95, 101]
TLR6	Human	Glioma tissues	Upregulated	Pro-	[55, 61, 62]
		Glioma cell lines (U87-MG, A172)	Upregulated	Pro-	[55, 59–62, 67, 68]
		Microglia	Upregulated	Anti-	[71, 72]
		GAMs	Upregulated	Pro-	[62, 75–78]
TLR7	Human	pDCs	Downregulated	Pro-	[82, 83]
		mDCs	Upregulated	Anti-	[69]
	Mouse	Glioma cell lines (GL261, CNS-)	Present	n.s	[69, 95, 98, 102]
TLR8	Mouse	Glioma cell lines (GL261, CNS-1)	Present	n.s	[69]
TLR9	Human	Glioma tissues	Upregulated	n.s	[55, 59, 60]
		Glioma cell lines (U87, LN229, SNB19, U251)	Upregulated	n.s	[59, 60, 79, 81, 103]
		Microglia	Upregulated	Anti-	[71, 79]
		pDCs	Downregulated	Pro-	[82, 83]
		GSCs	Upregulated	Pro-	[93]
	Murine	Glioma cell lines (GL261, C6)	Upregulated	n.s	[95, 98]
		Microglia	Upregulated	Anti-	[71, 79]
		GSCs	Upregulated	Pro-	[93]
TLR10	Human	n/a			
	Mouse	n/a			

n/a indicates that information is not available. *n.s.* not specified

TLR1 heterodimerizes with TLR2 in microglia of normal brains, and their expression increases in astrocytes and glial progenitors in respond to pathogen attack [71, 72]. Both in vitro and in vivo experiments have shown that TLR2 and TLR5 were highly expressed in tumor-infiltrating microglia and possessed antitumor activity. TLR2 actively responded to tumors cells and contribute to innate immune response by upregulating microglial major histocompatibility complex class I (MHC I). Such improved MHC I function and antigen-presenting system enhanced the accumulation, activation, and proliferation of CD8^+^ T-cells against tumor [72]. Activation or depletion of microglial TLR5, which specifically recognizes flagellin and usually associate with neurodegenerative disease, had no impact on the growth of murine GL261 gliomas. However, it was considered as modulator of microglial function by triggering their accumulation [73].

Glioma-derived factors can attract microglia and polarize them into pro-tumorigenic phenotype. Accumulation of glioma-associated microglia/macrophages (GAMs) at the tumor site shows compromised ability against tumor and usually associates with poor clinical prognosis [74]. Activation of TLR1/2 heterodimers and TLR2/6 heterodimers in GAMs plays an important role in extracellular matrix remodeling and tumor expansion through induction of matrix metalloproteinases (MMPs), interleukin (IL)-6, and inducible nitric oxide synthase (iNOS) [62, 75–77]. Moreover, expression of microglial MHC II is reduced in HGGs indicating diminished antigen-presenting ability of GAMs, which further hindered CD4^+^ T-cell activation at tumor site [78]. TLR2 knockout in glioma mouse model significantly reduced GAM accumulation and led to tumor regression and survival benefit [76].

In addition, combined activation of the endosomal TLR3 and TLR9 in microglia was shown to have synergistic effect both in vitro and in vivo, reinforcing the secretion of proinflammatory factors, phagocytic activity, and suppression of glioma growth. This could be further enhanced by combining with CD47 blockade [79]. These results indicate that TLRs expressed on microglia or GAMs could be used as potential targets for glioma treatment.

#### TLR expressions on dendritic cells

The antitumor immune response depends largely on antigen-presenting cells (APCs) such as DCs, which can be further categorized into plasmacytoid DC (pDCs) and myeloid DC (mDCs). Both types are known to modulate immune responses by reacting to TLR activation through cytokine secretion and antigen presentation to T and B lymphocytes, inducing cytotoxicity in tumor cells [80]. pDCs express high levels of TLR7/9 which upon recognition of viral DNA/RNA produce type I interferons (IFNs) and induce NK and macrophage activation (innate immune response) and T-cell expansion (adaptive immune response) to promote tumor cell lysis [81]. In glioma, pDCs exhibit an impaired response to TLR7/9 stimulation and are defective in T-cell immunity and type I IFN secretion, resulting in regulatory T-cell (Treg) accumulation and immunosuppressive microenvironment, which further contributes to tumor progression and poor patient survival [82]. Depletion of pDCs could increase the survival of glioma-bearing mice by reducing the number and suppressive ability of Tregs [83].

Some evidence suggests that both mDC infiltration and antitumor T-cell expansion in GBMs are TLR2-dependent [84]. In GBM-bearing mice, vaccination with mDCs resulted in type 1 T-helper (Th1) immune response and infiltration of CD4^+^ and CD8^+^ T-cells, leading to tumor elimination and prolonged survival [83]. TLR7 activation by imiquimod caused human DCs to become tumoricidal [85], possibly by triggering tumor-specific cytolytic T-cells and activation of antigen presentation. This led to subsequent tumor eradication and establishment of the immunological memory against secondary tumor cell transplants in mouse models [69].

Together, these results suggest that DCs may be targeted for potential immunotherapy, either through induction of cytokine production, disruption of immunosuppressive mechanisms, or stimulation of the antitumor microenvironment.

### TLR expressions on glioma stem cells

Cancer stem cells are the minorities among the bulk glioma cells but are well known to cause therapeutic resistance, tumor growth, and recurrence. In gliomas, TLRs are expressed by tumor cells and GSCs, contributing to a strong immunosuppressive microenvironment [86, 87].

It was reported that GSCs have significantly higher TLR2 expression than adherent GBM cells [88]. Administration of TLR2 agonist Pam3CSK4 or TLR4 agonist LPS markedly diminished the expression of GSC markers, implying a TLR-dependent differentiation; dual application of Pam3CSK4 and TMZ resulted in an increased GBM cell sensitivity to chemotherapy [86]. Pam3CSK4 increased the migratory and invasive capability of GSCs by enhancing MMP-2 and MMP-9 expression, while TLR2 knockout attenuated the effects and prolonged survival in glioma mouse model [88, 89].

**Fig. 3:**
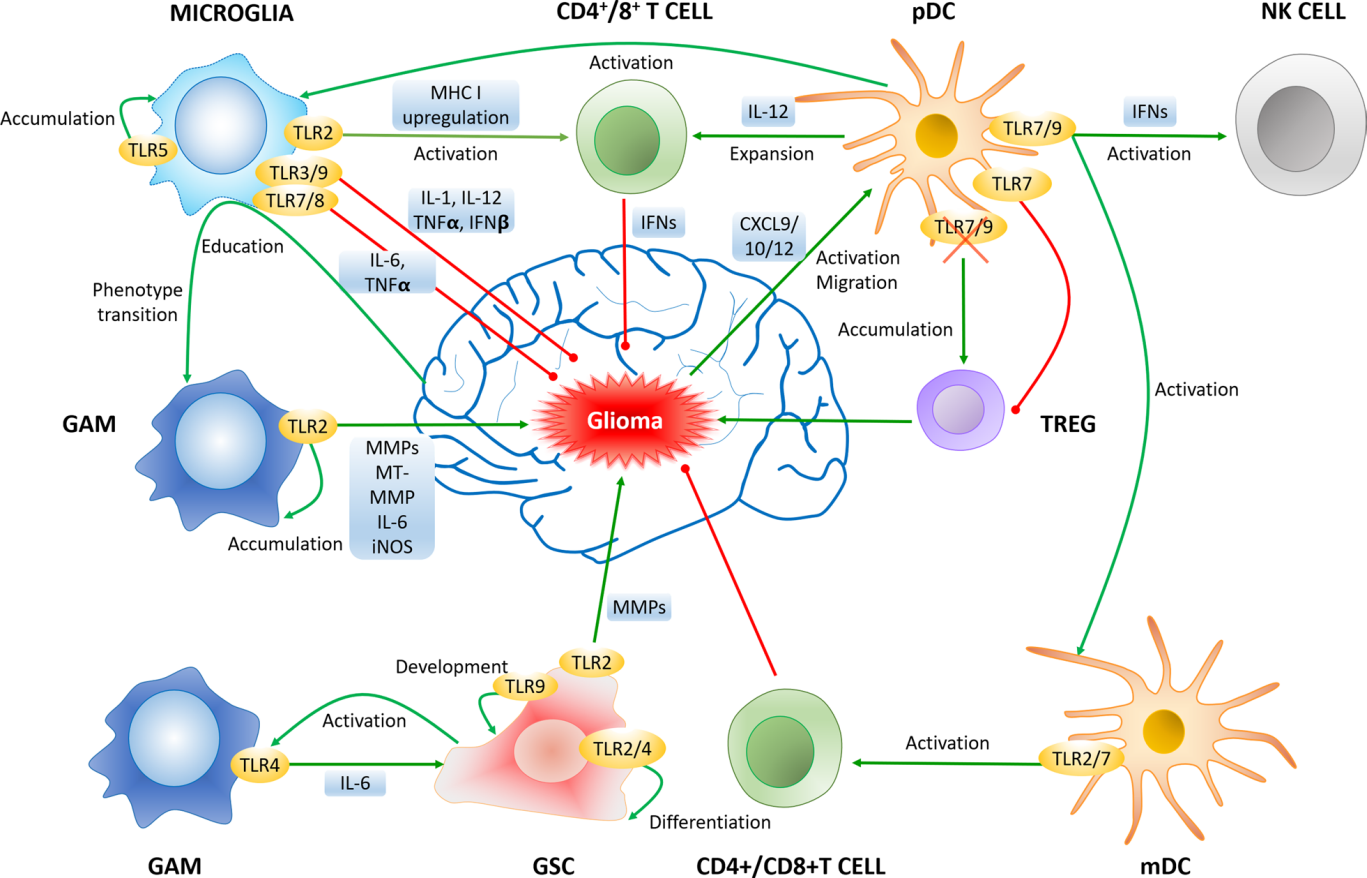
Cross talk between immune cells and glioma via TLRs in glioma TME. Activation of TLR3, 7, 8, and 9 on microglia inhibits glioma growth via secretion of pro-inflammatory cytokines such as ILs, TNFs, and IFNs. Agonists initiate TLR7/9 functions on pDCs to stimulate NK and microglia through IFN secretion and diminish infiltrating Treg, while blockade of TLR7/9 accumulates Treg and accelerate glioma progression. Activation and expansion of cytotoxic T-cells are stimulated by pDC and TLR2/7 expressed on mDC to exhibit antitumor activity. The tumor-infiltrating microglia can be educated by glioma to transit into protumor phenotype (GAM) and promote glioma invasion and progression through release of MMPs, ILs, and iNOS. TLR2, 3, and 4 are necessary for GSC differentiation or development. GSC activates TLR4-expressing GAMs, which release IL-6 that further promotes GSC development, forming a positive loop to support glioma development. The green and red arrows represent promotion and inhibition, respectively

GSCs have been reported to instigate immune suppression through TLR4 downregulation, allowing them to survive in the TME by abrogating inflammatory signals [90]. TLR4 overexpression decreased GSCs proliferation in xenografts, partially through suppressing the expression of core stem cell transcription factors (SOX2, NANOG, and OCT4) mediated by retinoblastoma-binding protein 5 (RBBP5) [90]. On the other hand, TLR4 downregulation ceased GSC growth by suppressing RBBP5 activity through TANK-binding kinase 1 (TBK1) phosphorylation in the TLR4-myeloid differentiation factor 88 (MyD88)-independent pathway [91]. During differentiation, GSCs upregulate TLR4 and release hyaluronic acid which acts as a TLR4 ligand, to further activate TLR4-NF-κB signaling pathway in a positive feedback loop. TLR4 blockade inhibited the NF-κB-mediated GSC proliferation [92]. Such inconsistency could be due to the location of TLR4 expression, where TLR4 overexpression on non-GSC cells tend to promote GSC proliferation. Moreover, cytosine–phosphate–guanosine oligodeoxynucleotide (CpG-ODN) was observed to activate TLR9 to promote the growth of GSCs through activation of signal transducer and activator of transcription 3 (STAT3) signaling in cultured cells, whereas silencing TLR9 expression abrogated the GSC development, suggesting TLR9 as a functional maker of GSCs and a target for therapeutic intervention [93]. GSCs also have cross talk with microglia and recruit GAM at glioma tumor site; GSCs-triggered GAMs release pro-inflammatory cytokine IL-6 via TLR4 signaling which is activated by GSCs-produced tenascin-C to promote glioma growth. TLR-4 or TLR adaptor protein MyD-88-deficient mice failed to secrete IL-6 [94] (Fig. 3).

## TLR signaling

All TLRs, with the exception of TLR3, either partly or fully depend upon the adaptor protein MyD88 for signaling activity [104]. Ligand binding to the LRRs in the ectodomain of TLRs affects the association of the cytoplasmic TIR domains and the MyD88. Activation of MyD88 results in myddosome formation by interacting with IL-1R-associated kinase-4 (IRAK-4), which then phosphorylates IRAK1 and IRAK2. This in turn promotes the activation of tumor necrosis factor receptor-associated factor 6 (TRAF-6) which acts as a ubiquitin ligase and triggers two major pathways. First, TRAF6 combines with ubiquitin-conjugating enzymes UBC13 and UEV1A and adds polyubiquitin chains to NF-κB essential modulator (NEMO) or IkappaB kinase gamma (IKKγ). IKKγ together with IKKα and IKKβ forms the IKK complex, which mediates rapid phosphorylation of IκB proteins, leading to release nuclear translocation and activation of NF-κB [105]. Second, TRAF6 activates the transforming growth factor β-activated kinase 1 (TAK1), which associates with two adaptor proteins: TAK1-binding proteins 1 (TAB1) and TAB2. TAK1 exerts double action: It phosphorylates IKKβ to activate NF-κB signaling or induces a cascade of mitogen-activated kinases (MAPKs) to promote phosphorylation of c-Jun kinases (JNKs), p38 MAPK, extracellular signal-regulated kinases 1 (Erk1) and Erk2, leading to activation of AP-1 transcription factor. These two signaling arms collaborate in controlling genes expression for inflammation mediators and extracellular matrix remodeling [43, 106].

In parallel, TLR activation also stimulates IFN signaling. NF-κB binds and upregulates the expression of the promoters of *interferon regulatory factor 1* (*IRF1*), *IRF2*, *IRF5*, and *IRF8* genes, forming a cross talk between the TLR and IFN pathways [107]. The transcription factor IRF1 directly associates with MyD88, IRF5 associates with both MyD88 and TRAF6, and TRAF6 also facilitates IRF7 in response to TLR7 and TLR9 activation. The IKK complex also participates in IRF3 and IRF7 activation, which induces type I IFN and host defense system. Ultimately, activation of the IRFs, NF-κB, and AP-1 transcription factors induces transcription of proinflammatory cytokine encoding genes, such as IL-6, IL-8, IL-18, IL-1β, TNFα, as well as PD-1 [108, 109].

**Fig. 4:**
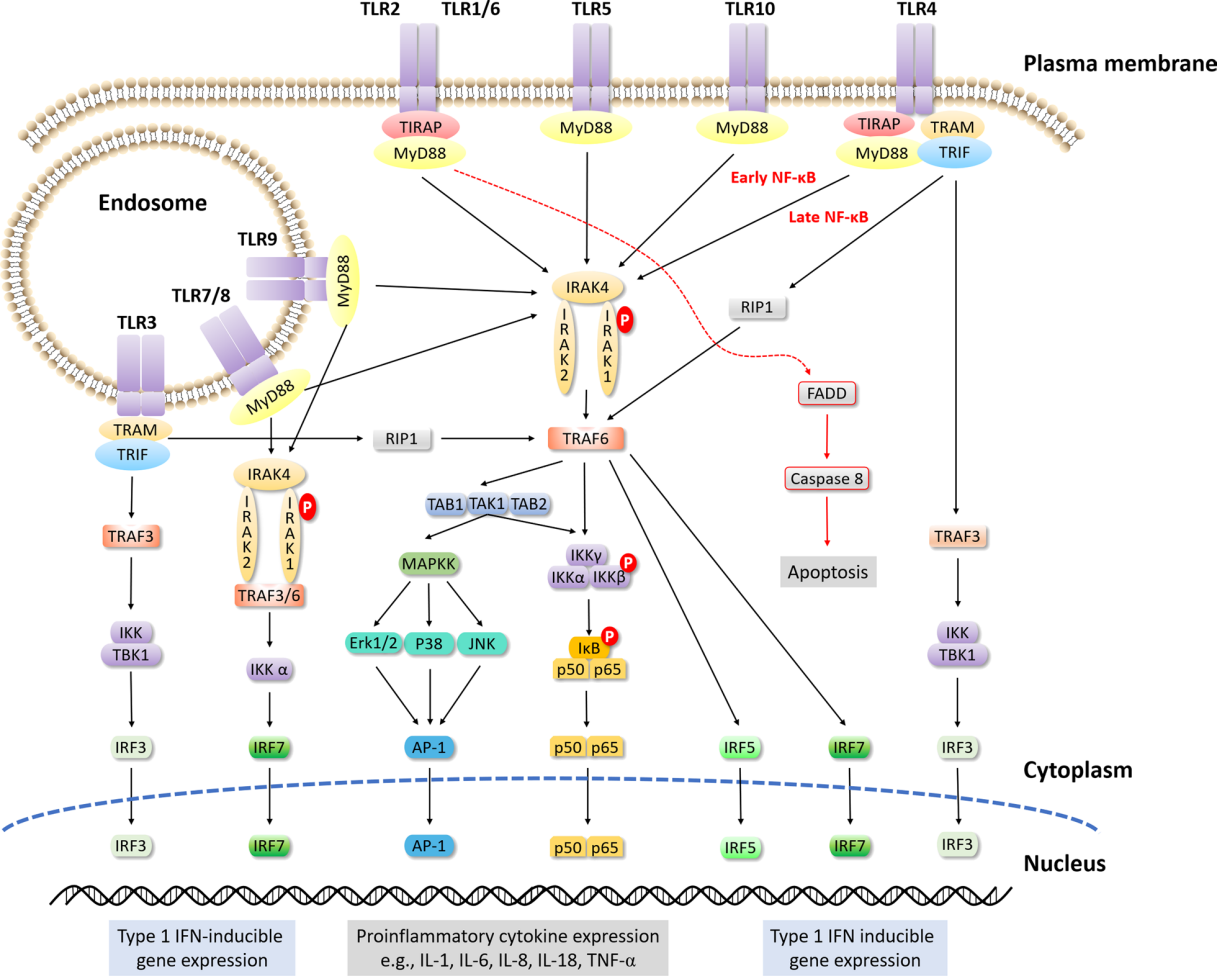
The TLR signaling pathways. TLRs are located on either plasma membrane or endosomal membranes where they detect PAMPs or DAMPs. TLRs trigger two main pathways: the MyD88-dependent and MyD88-independent pathways. The MyD88-dependent pathway is initiated after surface (TLR1/2, TLR2/6, TLR4, and TLR5) or intracellular TLRs (TLR7, TLR8, and TLR9) binding to corresponding ligands. Recruitment of MyD88 to the TIR domain of TLRs induces myddosome formation which involves MyD88, IRAK, and TRAF6. IRAK and TRAF6 stimulate TAK1 to activate IKKγ complex, which further releases NF-κB into the nucleus. The activated TAK1 also promotes MAPK activation which in turn stimulates AP-1 nucleus translocation. Both routes support proinflammatory cytokine transcription. In parallel, the myddosome also initiates IRF5 and IRF7 production to induce type I IFN gene expression. The MyD88-independent pathway is activated by TLR3 and TLR4. The TIR domain of TLRs recruits TRIF to form a complex containing TRAF3, TBK1, and IKK, which promotes nucleus translocation of IRF3, or initiation of a late phase NF-κB via interaction with RIP1 and subsequent TRAF6 activation. Both signaling stimulates the production of type I IFNs. The detailed signaling pathway is described in the text

Aside from MyD-dependent signaling, ligand binding to TLR3 and TLR4 also activates IRF3 through TIR-domain-containing adaptor-inducing interferon-β (TRIF) and TRAF3. TRAF3 subsequently induces IFN expression through TBK1. A second pathway mediated by TLR3 and TLR4 involves TRAF6 recruitment via interaction with receptor-interacting serine/threonine protein (RIP), which triggers a late phase of NF-κB signaling, initiating further production of proinflammatory molecules [58] (Fig. 4).

### TLR-mediated signaling in glioma

NF-κB signaling is a key driver of cell proliferation, migration, apoptosis, and immune responses through transcriptional regulation of gene expression. The NF-κB transcription factors consist of NF-κB1 p50, NF-κB2 p52, RELA (also named p65), RELB, and c-REL. The NF-κB gene expression signature has a prognostic value in glioma patients from the TCGA database. Nuclear expression of the p65 protein was proven to be an independent predictor of both OS and PFS in LGGs [110]. Nuclear localization of activated p65, which was prominent in microglia infiltrating LGGs, was found reduced in microglia of GBM tissues. The *IKBKB* gene (coding for IKKβ) expression and IKKβ levels were also found lower in GBM tissues compared with LGGs and were down-regulated in microglia infiltrating GBMs. The defective NF-κB signaling in innate immune cells infiltrating glioma was correlated with reduced expression of immune/inflammatory genes [111].

### TLR2 signaling in glioma

TLR2 heterodimerizes with TLR1 or TLR6 to induce signaling cascade upon recognition of a large variety of molecules such as microbial infectious ligands (peptidoglycan (PGN) and lipoprotein), viral ligands, signals generated from tissue injury (HSPs), and molecules released from necrotic cells (high-mobility group box 1 (HMGB1)). The MyD88-dependent TLR2 signaling activates NF-κB pathway, which in turn stimulates the transcription of proinflammatory cytokines and chemokines, and may lead to cellular proliferation and tumor progression [55, 112, 113].

TLR2 was found substantially elevated in glioma cell lines and tissues and inversely correlated with GBM patient survival [62]. The activation of TLR2 by agonist PGN was found to stimulate NF-κB signaling and increased cell growth [114]. TLR2 overexpression also enhanced cell autophagy and modulated p38/MAPK pathway, which in turn contributed to viability of glioma cells [61]. Upregulated endogenous TLR2 ligand versican was reported to induce the expression of membrane type 1 matrix-bound metalloproteinase (MT1-MMP) in GAMs which activates tumor-released MMP2 and of glioma cells, contributing to invasive and migratory behavior of glioma [115]. HSPs-expressed GAMs also contribute to glioma invasion through downregulation of MHC II molecules via MAPK/Erk1/2 signaling [78]. On the other hand, HMGB1 interacts with TLR2 on glioma cells to promote GSCs development and tumor progression via Wnt/β-catenin signaling, while their activation on DCs was shown to stimulate DC infiltration through activation of NF-κB, resulting in tumor suppression [83].

Overexpression of TLR2 was also observed in CNS inflammation and neurodegeneration such as multiple sclerosis (MS) and experimental autoimmune encephalomyelitis (EAE) [116]. TLR2 in conjunction with TLR4 and TLR7/8 signaling strongly suppresses type I IFN amplification and Treg function and promotes Th1 function against pathogen attack and Th17 responses to trigger autoimmunity [117].

### TLR4 signaling in glioma

TLR4 recognizes various exogenous PAMPS and endogenous ligands such as LPSs. Activation of TLR4 signaling occurs in both MyD88-dependent and MyD88-independent pathways and associates with regulation of tumor progression and immune evasion via production of cytokines, chemokines, and type I IFNs [64].

Elevated TLR4 expression was observed in glioma tissues and cell lines compared with normal brain tissues [118]. In glioma, activation of TLR4-mediated MyD88-dependent pathway was reported to correlate with upregulation of NF-κB signaling and increased expression of transcription factors (*JUN* and *SRF*)*,* implying promoted cellular proliferation [64]. Administration of TLR4 ligand LPS to glioma cells not only induced the activation of NF-κB pathway but also the MyD88-dependent Notch pathways, which suppressed the expression of glioma differentiation marker glial fibrillary acidic protein, leading to reversed glioma differentiation and tumor progression [87]. TLR4 was also shown as a key factor to promote NF-κB activation in GSC differentiation. Hyaluronic acid in the brain extracellular matrix has been described to trigger TLR4-NF-κB pathway in GBM stem-like cell differentiation and maintenance and consequently the tumorigenic capacity of GSCs [92].

In GBM, LPS stimulation may trigger late phase NF-κB (p65) nuclear translocation, suggesting MyD88-independent pathway activation [118]. In addition, TLR4 signaling in GBM may involve tumor suppressor *PTEN* which regulates TIRAP and TLR4 internalization [119]. *PTEN* loss/mutations which are frequently observed in high-grade *IDH* wild-type glioma can lead to decreased inhibition over TIRAP and consequent upregulation of MyD88-dependent NF-κB activation [55].

Furthermore, there is strong evidence that the expression of TLR4 interferes with Wnt signaling and apoptosis in glioma progression. TLR4 was overexpressed in human GBM cell lines and tissues, associating with down-expression of Dickkopf 3 (inhibitor of Wnt signaling pathway) and Claudin-5 (protein present in tight junctions), leading to induced Wnt/Claudin signaling to inhibit cell apoptosis and promote GBM progression [66]. TLR4 stimulation also correlates with suppression of apoptotic molecule caspase-9 to stimulate glioma progression [120]. Silencing TLR4 expression by RNA interference significantly abrogated GBM cell proliferation and upregulated apoptosis in vitro [66, 121]. This suggests that modulating TLR4-dependent Wnt signaling and apoptosis could be a potential therapeutic strategy in the treatment of gliomas.

Similar to TLR2, TLR4 participates in the pathogenesis of CNS inflammation MS by regulating autoimmune responses. LPS-induced TLR4 activation could stimulate NF-κB upregulation in Th1 and Th17 cells, leading to the secretion of proinflammatory cytokines IL-6 and IL-23, which in turn activates microglia to engage in neuroinflammatory reactions to kill neurons [122]. TLR4 expression by T-cells is essential for the development of EAE models of MS. Upon LPS stimulation, CD4^+^ T-cells exhibited enhanced survival, proliferation, and MHC II markers [123].

### TLR9 signaling in glioma

TLR9 is an intracellular TLR which senses for bacterial and viral DNA. In glioma, the expression of TLR9 correlates with malignancy [68]. STAT3 regulates TLR9 overexpression to maintain GSCs [124]. In addition, CCL2/CCL5 [125], MMP-2, and MMP-9 [126] have been implicated in TLR9-mediated glioma progression. MMP inhibition dramatically abolished TLR9-mediated GBM cell invasion in vitro [39]. Activation of TLR9 by agonist CpG-ODN could enhance GBM cell invasion [68]. On the other hand, it is also considered as a potential radiosensitizer to enhance the response of glioma cells to radiotherapy via TLR9 signaling. Activation of TLR9 upregulates NF-κB and MAPK signaling, leading to NO production, which is a key factor to increase the radiosensitivity of tumor through induction of apoptosis [127]. Moreover, in glioma cells, insulin-like growth factor 1 (IGF-1) could induce TLR9 expression through hypoxia-induced factor 1 alpha signaling, which further leads to secretion of cytokines IL-1β, IL-6, IL-8, and CXCR4 (a chemokine receptor that plays an important role in cell migration) [128].

The expression of TLR9 is also dramatically increased in neuroinflammation. CpG DNA potently induces IFN-α production in pDCs which overexpress TLR9 to further promote disease progression [129]. Up to date, the application of TLR9 agonist against neuroinflammations is conflicting as both suppressive and promoting results were observed [123]. The impact of TLR3, TLR5, TLR7, TLR8, and TLR10 signaling in glioma development is not fully elucidated.

## TLR agonists in glioma treatment

**Fig. 5:**
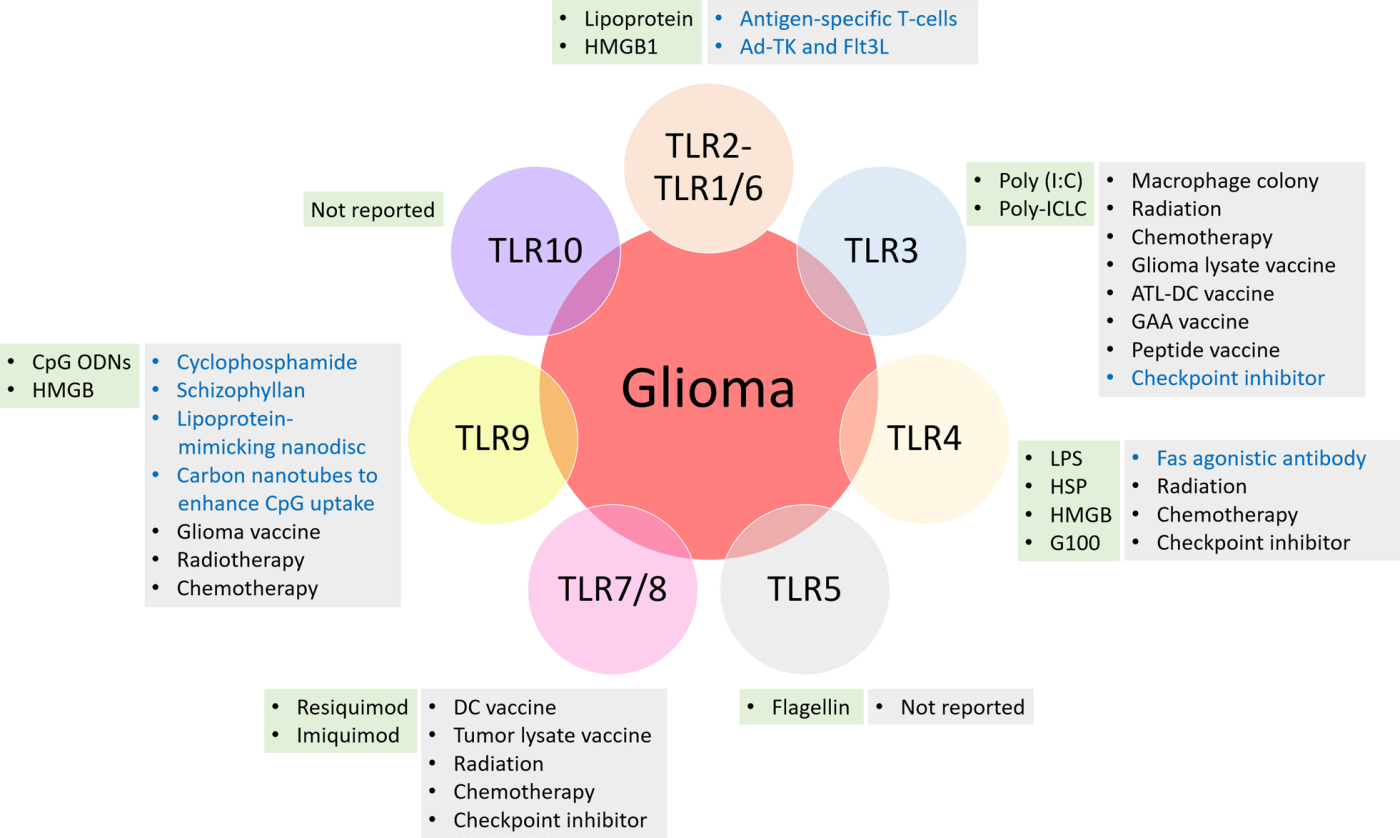
TLR agonists and combinatorial treatment. The green box represents TLR agonists, the gray box represents dual treatment with TLR agonists, the blue texts indicate in vivo or in vitro experiments, and the black texts indicate clinical trials

The expression patterns and signaling mechanisms make TLRs potential targets for glioma therapy, where multiple routes may be targeted to aid the development of effective clinical strategies. TLR agonists have been reported to initiate or suppress immune responses in the glioma environment upon binding to specific TLRs (Table 3). In particular, local administration of TLR agonists is of particular interest for immunotherapy (Fig. 5) (Table 4).

**Table 3:** TLR-based antitumor treatment in vivo or in vitro through agonist targeting

TLR	Agonist	Other treatment	In vitro cell line(s)	In vitro outcome(s)	In vivo model(s)	In vivo outcome(s)	Refs.
TLR1/TLR2	Pam3Cys-SK4	n/a	n/a	n/a	Murine GL261 implanted C57BL/6 J mouse	Survival benefit	[95]
	Lipoprotein	Antigen-specific T-cells	n/a	n/a	Murine GL261 implanted C57BL/6 J mouse	Survival benefit Immune memory development	[130]
TLR2	Pam3Cys-SK4	n/a	GSCs of G261	Increased GSC invasion	n/a	n/a	[88]
	Pam3Cys-SK4	n/a	U87, U118	GSC differentiation	n/a	n/a	[86]
	Pam3Cys-SK4	TMZ	U87, U118	GSC differentiation Increased cell death	n/a	n/a	[86]
TLR3	Poly (I:C)	n/a	n/a	n/a	Murine GL261 implanted C57BL/6 J mouse Rat CNS-1 implanted Lewis rats	Not effective	[69, 95]
	Poly (I:C)	n/a	n/a	n/a	Murine GL261 implanted C57BL/6 J mouse	DC-dependent survival benefit	[187]
	Poly (I:C)	Anti-PD-1	Primary human GBM cells	Immune activation T-cell attraction	Murine GL261 implanted C57BL/6 J mouse	Reinforced antitumor response Survival benefit Immune memory development	[186, 187]
	Poly (I:C)	T-cell vaccination	GL261	Ag-specific T-cells infiltration	Murine GL261 implanted C57BL/6 J mouse	Immune memory development	[214]
	Poly (ICLC)	Anti-PD-1	n/a	n/a	GL261 mouse glioma mode	Reinforced antitumor response Survival benefit	[137]
TLR3/TLR9	Poly (I:C) + CpG	n/a	Microglia of GL261, U87, LN229	Reduced tumor cell proliferation	Murine GL261 implanted C57BL/6 J mouse	Tumor suppression	[79]
	Poly (I:C) + CpG	CD47 blockade	Microglia of GL261, U87, LN229	Reduced tumor cell proliferation	Murine GL261 implanted C57BL/6 J mouse	Reinforced tumor suppression	[79]
TLR4	LPS	n/a	GSCs of patient-derived GBM xenografts	Inhibited non-GSC cell growth	Fresh human GBM cell implanted xenograft	Tumor regression	[90]
	LPS	n/a	U87, U118	GSC differentiation	n/a	n/a	[86]
	LPS	n/a	n/a	n/a	Murine GL261 implanted C57BL/6 mouse	Not effective	[95]
	LPS	n/a	n/a	n/a	Rat RG2 GSCs implanted Fisher 344 rats	Survival benefit	[100]
	LPS	n/a	n/a	n/a	Delayed brain tumor GBM cell implanted wild-type and Tlr-4 knockout BALB/c mice	Subcutaneous GBM injection: complete tumor removal Intracranial GBM injection: Wild-type: survival benefit	
	LPS	n/a	n/a	n/a	Patient-derived GBM cells implanted T lymphocyte-deficient nude mice	Complete tumor regression	[215]
	LPS	Fas agonist antibody	U118, U87	Reduced tumor cell proliferation	n/a	n/a	[151]
	G100	Tumor-specific T-cells	n/a	n/a	Murine GL261 cells-implanted C57BL/6, OT-I, PMEL, and B6.SJL mice	Complete tumor regression	[153]
TLR7	Aldara	n/a	GL261	Inhibited tumor cell proliferation (TLR7-independent)	Murine GL261-implanted C57BL/6 mouse	Inhibited tumor growth	[102]
TLR7/TLR8	R848	n/a	n/a	n/a	Murine GL261-implanted C57BL/6 mouse	Inhibited tumor growth Survival benefit	[95]
	R848	n/a	n/a	n/a	Rat CNS-1-implanted Lewis rats	Tumor regression Immune memory development	[69]
	R848	Cyclophosphamide	n/a	n/a	Rat CNS-1-implanted Lewis rat	Reinforced tumor regression Failed to reject secondary tumor challenge	[69]
TLR9	CpG ODN	n/a	GL261	Inhibited cell proliferation (TLR9-independent)	GL261-implanted C57BL/6 mouse	Inhibited tumor growth	[95]
	CpG ODN	n/a	GL261	Tumor cell apoptosis	GL261-implanted C57BL/6 mouse	Survival benefit	[171]
	CpG ODN	n/a	U87, U251, C6	Elevated invasion of U87 cells	n/a	n/a	[68]
	CpG ODN	n/a	n/a	n/a	Rat 9L and RG2-implanted Fisher 344 mice	Tumor remission in > 30% of the animals	[176]
	CpG ODN	Radiotherapy	n/a	n/a	Rat 9L and RG2-implanted Fisher 344 mice	Tumor remission in > 60% of the animals	[176]
	CpG ODN	Radiotherapy	U87, CHG-5, U251	Autophagy induction	Orthotopic tumor-bearing nude mice	Autophagosome formation	[177]
	CpG ODN	Radiotherapy	U87	Inhibited tumor cell proliferation	Human U87-implanted xenograft in nude mice	Tumor regression	[127]
	CpG ODN	Cyclophosphamide	n/a	n/a	Murine GL261-implanted C57BL/6NTac mice	Improved immune response Tumor regression Immune memory development	[175]
	CpG ODN	Glioma vaccine	GL261	Enhanced T-cell function	Murine GL261-implanted C57BL/6 mouse	Tumor regression Survival benefit	[190]
	CpG ODN	Schizophyllan	C6	Tumor cell apoptosis	n/a	n/a	[180]
	CpG	SWNT	K-Luc GL261	Inhibited tumor cell migration	n/a	n/a	[179]
	Docetaxel HDL-CpG	Radiation	GL26, CNS-1, HF2303, U251	GBM cell death	Murine GL261-implanted C57BL/6 mouse	Tumor regression Survival benefit Immune memory development	[181]

n/a indicates information that is not available

**Table 4:** The clinical trials TLR agonist-based treatment against glioma

Target	Agonist /antagonists	Combinatorial treatment	Indication(s)	Status	Phase(s)	Enrolment	Route	Response rate	Median PFS (months)	Median OS (months)	Number	Refs.
TLR2	Pam3Cys	None	GBM	Recruiting	I	15 A	*n.s*	n/a	n/a	n/a	NCT04842513	n/a
TLR3	Poly I:C	None	Gliomas Glioma	Completed	II	47 P/A	*i.m*	50% (LGG) 25% (HGG)	n/a	n/a	NCT01188096	[143]
	Poly I:C	Radiation	HGGs/GBM	Recruiting	I	30 A	*i.t*	20%	2.9	12	NCT03392545	[134]
	Poly I:C	Peptide vaccine	Recurrent LGGs	Completed	Early I	10 A	*s.c*	55.5%	12	n/a	NCT00874861	[140]
	Poly ICLC	n/a	Glioma HGG/GBM	Completed	II	55 A	*i.m*	11.1%	6moPFS 24%	11	NCT00058123	[138]
	Poly ICLC	GAAs	Grade II Astrocytomas	Completed	Early I	13 A	*i.m*	90.1%	17	n/a	NCT00795457	[140]
	Poly ICLC	GAAs	Recurrent HGGs	Completed	I	26 P	*i.m*	25%	4.1	12.9	NCT01130077	[145]
	Poly ICLC	GAAs	LGG	Completed	I	14 P	*i.m*	41.6%	9.9	42	NCT01130077	[144]
	Poly ICLC	GAAs	LGG	Recruiting	I	60 P/A	*i.m*	41.6%	9.9	42	NCT01130077	[144]
	Poly ICLC	Peptide vaccine TMZ	Astrocytoma GBM	Completed	I/II	19 A	*i.m*	31.2%	9.5 for GBM	19 for GBM	NCT01920191	[141]
	Poly ICLC	APVAC1 vaccine	GBM	Completed	I	16 A	*s.c*	n/a	14.2	29	NCT02149225	[37]
	Poly ICLC	Radiation TMZ	Newly diagnosed GBM	Completed	II	97 A	*i.m*	n/a	n/a-	15	NCT00262730	[216]
	Poly ICLC	GAAs	Recurrent malignant glioma	Completed	I/II	22 A	*i.m*	n/a	n/a	n/a	NCT00766753	n/a
	Poly ICLC	SL-701	Recurrent GBM	Completed	I/II	74 A	*i.m*	n/a-	n/a-	n/a-	NCT02078648	n/a-
	Poly ICLC	GAAs	LGG	Recruiting	II	25A	*i.m*	n/a-	n/a-	n/a-	NCT02358187	n/a-
	Poly ICLC	Peptide vaccine Pembrolizumab	Recurrent Relapsing GBM	Recruiting	I/II	24 A	*s.c*	n/a-	n/a-	n/a-	NCT03665545	n/a-
	Poly ICLC	Glioma lysate vaccine	Grade II2 glioma	Recruiting	I	30 A	*s.c*	n/a-	n/a-	n/a-	NCT02549833	n/a-
	Poly ICLC	K27M peptide Nivolumab	Newly diagnosed glioma	Recruiting	I/II	49 A	*n.s*	n/a	n/a	n/a-	NCT02960230	
TLR4	HSPPC-96	None	Recurrent GBMGlioma	Completed	I/II	41 A	*n.s*	n/a	19.1 weeks	42.6 weeks	NCT00293423	[158]
	HSPPC-96	None	Newly diagnosed GBMGlioma	Completed	I	20 A	*n.s*	20%	11	31.4	NCT02122822	[157]
	HSPPC-96	Temozolomide	Newly diagnosed GBM	Completed	II	70 A	*i.d*	26%	18	23.8	NCT00905060	[189]
	HSPPC-96	Bevacizumab	GBMRecurrent GBM	Active not recruiting	II	90 A	*i.d*	n/a	n/a	n/a	NCT01814813	n/a
	HSPPC-96	Pembrolizumab Temozolomide Radiation Placebo	Newly diagnosed GBM	Active not recruiting	II	310 A	*i.d*	n/a	n/a	n/a	NCT03018288	n/a
	Ibudilast	TMZ	Recurrent GBM	Active not recruiting	I/II	50 A	*n.s*	n/a	n/a	n/a	NCT03782415	n/a
TLR7	Imiquimod	DC vaccine	Recurrent malignant brain tumor	Completed	I	8 P/A	*s.a*	n/a	n/a	n/a	NCT01171469	n/a
	Imiquimod	DC vaccine	Malignant glioma	Completed	I	71 A	*s.a*	n/a	n/a	n/a	NCT01792505	n/a
	Imiquimod	Tumor lysate vaccine	Recurrent grade II glioma	Completed	Early phase I	19 A	*n.s*	n/a	n/a	n/a	NCT01678352	n/a
	Imiquimod	hP1A8 GBM6-AD vaccine	Recurrent GBM	Recruiting	I	24 A	*n.s*	n/a	n/a	n/a	NCT04642937	n/a
	Imiquimod	IDH1R132H-specific vaccine Avelumab	Progressive diffuse glioma	Recruiting	I	60 A	*n.s*	n/a	n/a	n/a	NCT03893903	n/a
	Imiquimod	DC vaccine Tumor lysate	Malignant glioma and GBM	Active not recruiting	I	20 A	s.a	n/a	n/a	n/a	NCT01808820	[169]
	Resiquimod	Adjuvant Poly ICLC Tumor-lysate pulsed DC vaccine	Brain tumors	Active, not recruiting	II	60 A	s.a	n/a	n/a	n/a	NCT01204684	n/a
TLR9	CpG-ODN	None	Malignant GBM	Completed	II	80 A	*n.s*	n/a	9	n/a	NCT00190424	[174]
	CpG-ODN	None	Recurrent GBM	Completed	II	n/a	*n.s*	n/a	28 weeks	n/a	n/a	[172]

n/a indicates information that is not available. *n.s.*, not specified; *i.m.*, intra-muscle; *i.t.* intra-tumorem; *i.d.* intra-derma; *s.c.,* sub-cutem; *s.a.*; skin absorption. *All clinical trials listed on http://clinicaltrials.gov/ at the date of submission

### TLR1/2 agonists for glioma treatment

In GBM-bearing mice, dual treatment with TLR1/2 agonist lipoprotein and adoptively transferred antigen-specific T-cells improved survival and restored immune protection. This antitumor immunity was achieved through modulation of the TME by maintaining the antitumor efficacy of adoptive T-cells, upregulating IFN-γ-secreting CD8^+^ T-cells and downregulating myeloid-derived suppressor cells (MDSCs) [130]. Although the study in animal model has shown some promising results, no clinical information is yet available on the efficacy of lipoprotein against gliomas. HMGB1 released from dying glioma cells works as TLR2 agonist on DCs to promote DC migration, and subsequently prime CD8^+^ T-cell cytotoxic immune response. The TLR2-mediated activation of DCs did not cause brain toxicity or autoimmunity in glioma-bearing mice, which is important for the effectiveness of gene/immunotherapy. The absence of TLR2 from DCs significantly eliminated the efficiency of gene/immunotherapy and resulted in exacerbated GBM tumor burden in mice [131].

### TLR3 agonists for glioma treatment

#### Poly I:C

TLR3 ligands serve as natural inducers of pro-inflammatory cytokines and type I IFNs that are capable to promote immune response and are among the most intensively studied TLR agonists against glioma. TLR3 ligands polyinosinic–polycytidylic acid (Poly I:C) is well known for its immunostimulatory activity, such as promoting the activation of DCs to prime antigen-specific T-cell function [132] and enhancing macrophage transition into antitumor phenotype when combined with proprotein convertases inhibitor, also known as macrophage’s reactivation drug [133].

In recent years, the effects of combining standard treatment with immune adjuvants have been assessed in cancer immunotherapy. Intratumoral administration of Poly I:C and granulocyte macrophage colony were applied before and after radiation therapy in recurrent grade IV glioma patients (NCT03392545) and showed 20% of response rate with median PFS and OS to be 2.9 and 12 months, respectively [134]. However, a few studies reported Poly I:C as stimulator for cancer cell migration [135], suggesting that extra caution is required in application.

#### Poly ICLC

Another well-known TLR3 agonist, polyinosinic–polycytidylic acid, and poly-L-lysine (Poly ICLC) or Hiltonol, is a double-stranded RNA complex to activate immune cells and works as potent vaccine adjuvant with broad innate and adaptive immune-enhancing effects [136]. A combination of Poly ICLC with radiation therapy was administered with anti-PD1 to GBM-bearing mice and showed enhanced antitumor effects of radiotherapy by inducing the cytotoxic activity of both cytotoxic T lymphocytes and NK cells [137].

A number of clinical studies have been performed in the last decade to evaluate the efficiency of Poly ICLC, either as stand-alone or in combination with other treatments against glioma.

Poly ICLC was intramuscularly administrated in most clinical trials and subcutaneously in others and was generally well tolerated (NCT00058123 and NABTC01-05) [138, 139].

Trials of Poly ICLC combined with peptide vaccination showed induction of potent T-cell responses in glioma patients. Glioma-associated antigens (GAAs) overexpressed in brain tumors have been used for vaccination in addition to Poly ICLC to treat glioma [140]. Elevated GAA-specific T-cell responses were observed (NCT00795457), and LGG patients without disease progression showed significantly higher IFN-γ production and better PFS (17 months) than recurrent patients (12 months) [140]. Application of Poly ICLC with multi-tumor-associated peptides vaccine IMA950 led to strong T-cell response and PFS and OS of 9.5 and 19 months, respectively, for GBM patients (NCT01920191) [141, 142]. However, Poly-ICLC-IMA950 vaccine did not improve the response to subsequent treatment of commonly used anti-angiogenic agent bevacizumab. The toxicity of the treatment was not reported [141]. Interestingly, T-cell responses were found absent in GBM patients on dexamethasone—a common corticosteroid prescribed to treat cerebral edema in GBM—during neoantigen-targeting vaccination and should be taken into account in future trial design [36].

A recent phase I trial (NCT02149225) applied more personalized immune vaccination in newly diagnosed GBM patients. Personalized peptides were designed based on individual tumor mutation and transcriptome/immunopeptidome data to serve as tumor-specific antigens. The peptides were administrated with Poly ICLC and standard care. Positive safety and immunogenicity results were achieved with median PFS and OS of 14.2 and 29 months, respectively. This is much beneficial than other treatment under similar conditions [37], showing a great potential for future brain tumor treatment. Other trials that are recruiting patients include the evaluation of Poly ICLC with glioma lysate vaccine to treat LGG (NCT02549833) and with ATL-DC vaccine/pembrolizumab (monoclonal antibody) to treat GBM (NCT04201873).

Other than vaccination, clinical trials have been performed to evaluate the efficiency of Poly ICLC in combination with concurrent therapies. Beneficial results were achieved when Poly ICLC was combined with landmark TMZ and radiation in newly diagnosed GBM, which gave a PFS of 17.2 months (NCT00262730), longer than the contemporary reference of 14.6 months.

The efficiency of Poly ICLC was also evaluated in children, given that pediatric LGGs have high risk to undergo malignant transformation into HGG, which usually have exceedingly poor prognosis with current therapies. Administration of Poly ICLC to children with various brain tumor subtypes was performed in a phase II trial (NCT01188096) [143], resulted in response rate of 50% (5/10) in LGGs and 25% (3/12) in HGGs, with no severe toxicity detected [143]. Administration of GAA vaccination with Poly ICLC showed median PFS and OS of 9.9 and 42 months in LGG patients (NCT01130077) [144] and 4.1 and 12.9 months in HGG patients [145], respectively, with reasonably good drug toleration. The less encouraging results may reflect the differences in the protein expression profile and immune milieu in the pediatric patients [145]. Prompt cytotoxic T-cell response was observed in an early phase I trial involved in the application of peptide-based vaccine and Poly ICLC in LGG children [146].

Although TLR3 agonists may not work as efficient drugs against glioma alone, they serve as adjuvant immune modulators to enhance the efficacy of vaccination or concurrent treatment against gliomas, especially with personalized immune vaccination. The presence of Poly ICLC could aid infiltration of immune cells into tumor site, which is usually inadequate in “immune-cold tumors” such as GBM [147]. Given that clinical studies using poly ICLC to treat gliomas have yield much less pronounced results than observed in animal models, a key factor may lie in the locality of administration. In most clinical trials, Poly ICLC was applied either intramuscularly or subcutaneously, whereas in animal models intratumoral application was more common. In addition, the lack of glioma hallmarks and surgical process in classical in vivo glioma models do not necessarily represent the complexities of human gliomas. More sophisticated design of preclinical manipulations may provide more accurate guidance to clinical translation.

### TLR4 agonists/antagonists for glioma treatment

#### LPS

LPS-induced TLR4 activation plays dual roles in glioma progression: It stimulates TLR4 signaling and promotes GBM pathogenicity through tumor cell proliferation and migration [148], while induces antitumor effects by eradicating GSCs [100] or causes macrophage infiltration and leads to tumor reduction and prolonged survival in GBM-bearing mice [149]. Differences in the actions may correlate with IL-17 interaction, which acts as pro- or anti-cytokine depending on the investigated neoplasm model [150]. Interestingly, simultaneous activation of TLR4 and Fas pathway resulted in the disappearance of TLR4- or Fas-induced tumor-promoting properties through reduction of MMP-9 expression [151], providing a potential TLR-based therapeutic option. LPS-mediated TLR4 signaling was also associated with immune phenotype transition in GBM cells and GSCs to induce antitumoral effect, leading to better survival in a cohort of patients with GBM [152]. However, no clinical trial of LPS application against glioma has been reported yet.

#### HSP

HSPs function as intracellular chaperons and are associated with the innate and adaptive immune system [153]. T-cell-supported HSP stimulation on APCs initiates NF-κB activation and has cross talk with TLR4 signaling, leading to release of inflammatory factors and attenuation of GBM growth. In GBM cells, HSP–peptide complexes reportedly interfere with TLR2 and TLR4 [154]. HSP90α favored GBM cell migration through TLR4-mediated EGFR induction and elevation of cytosolic Ca^2+^ [155]; HSP60 was reported as a tumor promotor through interaction with TLR4 in glioma cells [156].

HSPPC-96 produced from patients’ tumor cells has been the most studied HPS–peptide vaccine against glioma. Administration of HSPPC-96 alone showed median PFS of 11 months and OS of 31.4 months in newly diagnosed GBM without severe adverse effects (NCT02122822) [157]. In patients with recurrent GMB, the OS was only 42.6 weeks, and patients with significant lymphopenia prior to treatment had even poorer immunotherapy response (NCT00293423) [158]. Further design of phase II trial (NCT00905060) was performed to compare the efficiency of HSPPC-96 with standard treatment (surgical resection, TMZ, and radiation) in GBM patients; improved PFS (18 months) and OS (23.8 months) were observed with tolerable toxicity. Trials comparing results with other immunological agents (pembrolizumab, bevacizumab) are also under evaluation (NCT03018288, NCT01814813).

#### HMGB1

Another application of TLR4 signaling concerns HMGB1 which plays an important role in mediating glioma cell proliferation, survival, and migration [159]. In vivo studies showed that secretion of HMGB1 by dead glioma cells during antitumor treatment stimulates TLR4 signaling and leads to DC maturation, tumor antigen presentation, and subsequent GBM regression [160, 161]. HMGB1-triggerred TLR4-dependent inflammatory responses promoted cytokine secretion and tumor development [63] and resulted in spontaneous epithelial–mesenchymal transition and invasion in GBM cells through overexpression of TLR4-signaling-regulated p62 [162]. Blocking TLR4-HMGB1 interaction by β-defensin could well suppress the proinflammatory microenvironment [63].

#### Synthetic TLR4 agonists and antagonists

Discovery of new small molecules that modulate TLR4 activity as agonists or antagonists has been under investigation for drug development. Synthetic glucopyranosyl lipid A (G100) was developed as TLR4 agonist to enhance antigen presentation to induce T-cell inflammation at TME [163]. Promising results were obtained in A20 lymphomas murine model where tumor cell apoptosis was observed both in vitro and in vivo [163]. Other TLR4 agonist includes monophosphoryl lipid A and trehalose derivatives [164]. However, clinical trial of these agonists in glioma has not been reported.

TLR4 antagonist ibudilast is an inhibitor of macrophage migration inhibitory factor and phosphodiesterases-4. The application of ibudilast was reported to possess anti-inflammatory capacity by reducing pro-tumor TLR4-downsignaling via apoptosis and suppression of GBM cell proliferation [165]. Clinical trial evaluating Ibudilast and TMZ combo treatment against recurrent GBM (NCT03782415) is under recruiting status. Other TLR4 antagonists that are yet to be studied in glioma treatment include FP compounds (HMGB1 competitor), IAXO compound (LPS competitor), and non-competitive inhibitor TAK-242 [164].

### TLR7/8 agonists for glioma treatment

#### Resiquimod

TLR7 and TLR8 are structurally and functionally relevant TLRs that trigger cytokine and IFN responses. The activation of TLR7/8 by agonist induces antitumoral immune response with high specificity [166]. In glioma-bearing mouse, TLR7/8 agonist R848 (resiquimod) has been shown to effectively eradicate tumor cells through T-cell proliferation, allowing the development of immunological memory and rejection of secondary tumor after re-challenge [69]. However, it remains to be clarified whether this antitumor activity was indeed TLR7/8-dependent.

#### Imiquimod

Another TLR7/8 agonist imiquimod (Aldara) is an FDA-approved immune response modifier. Imiquimod was shown to inhibit glioma cell proliferation and prolong the survival of intracranial glioma-bearing mice through reduced CD4^+^FoxP3^+^ Treg cells and increased tumor-infiltrating DCs, CD4^+^, and CD8^+^ T-cells [102]. In addition, imiquimod was reported to initiate Th1 immune response by activating tumor sentinel cells and repressing Hedgehog signaling through negative modulation of glioma-associated oncogenes in GL261 cells [167].

In cancer vaccine therapies, imiquimod together with TLR3 agonist Poly I:C/Poly-ICLC was considered as immune adjuvants to enhance DC maturation and antitumor T-cell responses[168]. In general, Poly I:C/Poly-ICLC was injected intramuscularly or subcutaneously, while imiquimod cream was administrated on skin before and/or after each vaccination [132]. Imiquimod cream combined with tumor lysate or DC vaccine was studied to treat malignant glioma after complete tumor resection (NCT01792505, NCT01171469, and NCT01678352). Efficient antitumor-associated antigen-specific CD4 + and/or CD8 + T-cell response and Treg depletion were induced in GBM patients treated with vaccination, imiquimod, and Poly ICLC and resulted in favored OS compared with those who received standard treatment (NCT00068510, NCT03893903) [132, 169]. However, small sample size should be considered as a limitation before drawing conclusion. Further trials will be performed on glioma patients with unfavorable molecular profiles to evaluate the efficiency of IDH1R132H-specific vaccine with imiquimod and checkpoint inhibitor avelumab (NCT03893903).

Unfortunately, TLR7/8 agonists also have issues that must be overcome prior to broad clinical implementation, such as systemic toxic effects. A terminated phase I trial (NCT01400672) with 8 children and young adult participants (3–25 years) exhibited strong toxic effects when tumor lysate vaccine was combined with imiquimod and radiation to treat brain stem glioma and GBM. Moreover, development of pro-drug-based delivery platform may be required for TLR7/8 agonists to enhance robust local activity and drug solubility [170].

### TLR9 agonist

#### CpG-ODNs

TLR9 recognizes foreign unmethylated CpG DNA to induce Th1 response and immunity. TLR9 agonists CpG-ODNs are considered strong activators of the innate and adaptive immunity. In vitro and in vivo studies showed that CpG-ODN-induced TLR9 activation increased cytokine expression to stimulate NK and T-cell responses, promoted tumor cell apoptosis, and prolonged the survival of GBM mouse models [95, 103, 171]. A single intratumoral injection of CpG-ODN could effectively inhibit glioma growth in 80% of glioma-bearing mice [95].

In clinical trials, intracranial CpG-ODN administration did not show favored OS and PFS in recurrent GBM patients [172]. CpG-28 was well tolerated at dose up to 0.3 mg/kg subcutaneously and 18 mg intratumorally; however, poor effectiveness was observed in glioma patients [173]. More recently, a phase II trial indicated that injection of CpG-ODN to surgical cavity of GBM patients after tumor removal followed by standard of care resulted in increased 2-year survival rate (31% vs 26%) and median PFS (9 vs 8.5 months), as compared with applying standard of care alone (NCT00190424) [174]. Nevertheless, clinical trials with CpG-ODN as a single agent demonstrated insufficient efficacy to treat majority of patients with high tumor burden.

CpG-ODN was shown to be more effective when combined with other treatments to enhance the immune-based antitumor response in vitro and in vivo. In preclinical glioma models, CpG-1826 combined with cyclophosphamide treatment increased the accumulation of GAMs, DCs, B-cells, and cytotoxic T-cells and led to long-term tumor regression accompanied by immune memory [175]; intratumoral injection of CpG-ODN was also advantageously associated with radiotherapy to induce complete tumor remission [176]. In vitro studies showed that CpG-ODN107 in combination with radiotherapy could stimulate autophagic glioma cell death via the TLR9-Erk-mTOR signaling pathway [177] and exerted a radio-sensitizing effect through TLR9-mediated NF-κB activation and NO production in tumor cells, leading to cell cycle arrest [127].

Moreover, the development of nanoparticles has presented a promising strategy to enhance drug delivery and immune response in glioma treatment [178]. Single-walled carbon nanotubes non-covalently functionalized with CpG were reported to activate macrophages through induction of the TLR9-NF-κB pathway and inhibit glioma cell migration [179]. Schizophyllan, a polymer that protects short DNA from endosomal degradation, efficiently enhanced CpG-ODN delivery across the blood–brain barrier to target TLR9 on immune cells and repolarized GAMs to the antitumor phenotype by inducing high levels of inflammatory cytokines [180]. A synthetic high-density lipoprotein-mimicking nanodisc was reported to deliver CpG with docetaxel (chemotherapeutic agent) to elicit CD8^+^ T-cell infiltration in glioma models and resulted in long-term survival and development of anti-GBM immunological memory when combined with radiotherapy [181].

Up to date, injection of CpG into glioma tumors showed promise as an immunotherapy in mouse models but proved disappointing results in human trials, suggesting the importance to apply novel combinatorial strategies into clinical trials. In a recent phase I study (NSC 733972), an IL-12 expressing Herpes Simplex virus M032, which contains rich unmethylated CpG, will be intracranially administrated to HGG patients together with checkpoint inhibitor after its safety is tested in animal models in pursuit of novel therapy for malignant glioma.

## TLR-based immune checkpoint inhibitor treatment

Immune checkpoints are known as double-edged swords for their ability to protect against autoimmunity, while also providing a path for immune escape in tumorigenesis. PD-1 and PD- PD-L1 have been implicated in tumor invasion by impairing antitumor response through exhaustion of effect CD8^+^ T-cells and NK cells [182]. In GBM, the expression of PD-L1 on tumor tissue promotes PD-1 activation in microglia that leads to diminished T-cell response [183]. Up to date, checkpoint blockade monotherapy is effective only in a small fraction of patients with glioma due to reasons such as low levels of *PD-L1* expression, especially in *IDH*-mutant glioma, and exhausted tumor infiltrating T-cells [184, 185], and the results were merely comparable with standard TMZ and radiotherapy (NCT02337491, NCT02617589). Therefore, combination strategies are urgently required to unlock further therapeutic responses.

**Fig. 6:**
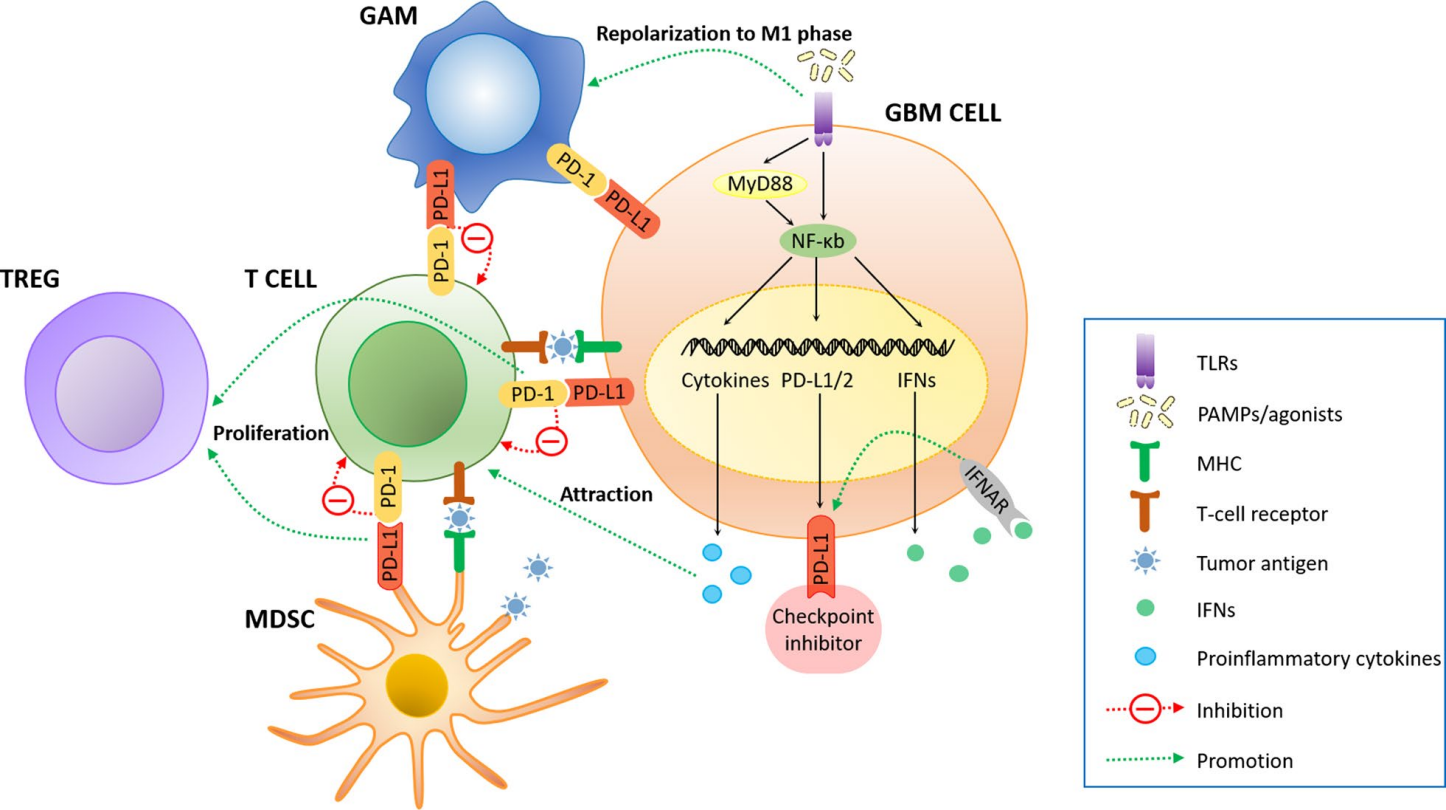
The TLR activation and induction of PD-L1/PL-D expression in GBM cell. In GBM TME, the PD-1/PD-L1 axis promotes tumor invasion through infiltration of MDSCs and Treg and exhaustion of cytotoxic T-cells. TLR recognizes ligand to elevate PD-L1 expression in three ways, the early-phase MyD-dependent NF-κB signaling, the late-phase MyD-independent NF-κB signaling, and IFN secretion which activates IFNAR signaling to induce further expression of PD-L1. TLR agonist combined with checkpoint inhibitor potentiates antitumor response by T-cell attraction, IFN production, PD-L1 upregulation and blockade of PD-1/PD-L1 axis

TLR activation affects PD-L1 expression in two ways: The MyD88-dependent signaling pathway promotes NF-κB-mediated cytokine transcription, PD-L1 gene transcription, and secretion; and the MyD88-independent cascade leads to late-phase NF-κB activation and type I IFN secretion, which not only enhances cancer immunotherapy, but also upregulates PD-L1 expression on tumor cells through interferon-α/β receptor (IFNAR) signaling [183] (Fig. 6). This suggests that PD-L1-expressed cells can be beneficial for TLR-based therapies in glioma and other cancer types.

Poly I:C-induced TLR3 activation has been reported to prime TME by stimulating PD-L1 and PD-L2 expressions on GBM cells, along with the secretion of proinflammatory cytokines such as IFNs, CXCLs and CCLs to attract CD4^+^ and CD8^+^ T-cell infiltration. This immune response can be effectively reinforced via blockade of elevated tumoral PD-L1 [186]. TLR3 activation also effectively promoted DC maturation and T-cell proliferation to enhance the antitumor response to anti-PD-1, resulted in increased survival rate in GBM mouse models [187].

Similarly, TLR4 overexpression was shown to promote PD-L1 expression in GBM patients via the MyD88-independent pathway, leading to autocrine induction of regional immunosuppression and poor prognosis. LPS, HMGB1, and HSPs produced in the GBM microenvironment which act as TLR4 agonists and further initiate signaling to promote PD-L1 expression [188]. High expression of PD-L1 on myeloid cells resulted in shorter OS in GBM patients treated with the HSP peptide vaccine [189]. In glioma-bearing mice, a mixture of DCs, CpG-ODN, and GSC lysate as a source of GSC-associated antigens was applied as vaccine (STDENVANT) to upregulate the expressions of PD-1 and PD-L1 on effector T-cells, DCs, and glioma tissues, leading to enhanced antigen presenting. Combining STDENVANT and anti-PD-L1 antibody attenuated Treg accumulation in the brain and showed an even greater survival advantage [190]. The results suggest that combining TLR activation to boost PD-L1 expression, alongside checkpoint blockade, provides a potential therapeutic benefit in glioma treatment. However, sufficient clinical studies are required to confirm its efficiency and safety in human.

Although limited number of studies are available on TLR immunotherapy against glioma, more results have been obtained from other cancer types and may be used as references. For example, in mice bearing melanoma, activation of TLR1/TLR2 dimer on APCs by agonist diprovocim was reported to induce strong cytotoxic T lymphocytes response to co-administered immune checkpoint inhibitor. This was mediated by proinflammatory cytokine production, antigen-specific eradication of tumor cells, in addition to anti-PD-L1 which further permitted antitumor T-cell activation [191]. In vivo study showed that intratumoral treatment of Poly I:C complexed with polyethylenimine (BO-112, to induce cancer cell apoptosis)-controlled tumor progression in melanoma-bearing mice along with increased PD-1/PD-L1 and CD137 expression. Combinatorial treatment of Poly I:C, BO-112, and checkpoint inhibitors (anti-PD-L1 and anti-CD137 monoclonal antibodies) enhanced the survival benefits through elevation of CD8^+^ T lymphocyte infiltration to the TME [192]. TLR4 expression was closely associated with PD-L1 expression in various cancer types [193–195]. TLR4-HSP86 axis was reported to induce T-cell PD-L1 expression and mediated tumor-cell-derived extracellular vesicle-generated MDSCs to suppress immune response. Disruption of the TLR4 signaling reduced MDSC in the TME and PD-L1 expression [195]. In non-small lung cancer patients, although no correlation between TLR7 and PD-L1 expression was observed, high TLR7 expression was closely associated with poor clinical response to immune checkpoint inhibitors and resulted in worse PFS and OS [196]. TLR7-dependent overexpression of PD-L1 was reported in monocytes treated with chronic lymphocytic leukemia-derived exosomes followed by production of various proinflammatory cytokines and chemokines [197]. Although TLR7 blockade with immunotherapy shows a therapeutic potential, it is important to be aware that TLR7/8 activation can also induce immunosuppressive MDSC via NF-κB signaling; therefore, inhibition of MDSC is critical for TLR7/8 agonist-based immunotherapy. Both in vitro and in vivo studies in urothelial carcinoma and melanoma showed that triple combination of TLR7/8-based nanovaccine, anti-PD-L1 antibody, and sunitinib (to reduce MDSCs/Tregs) significantly enhanced T-cell response and improved therapeutic efficacy [198].

As TLRs elicit both pro- and antitumor effects, understanding its functional regulations in tumor cells is crucial for the success of TLR-based immunotherapy. More clinical information of TLR-based immunotherapy in multiple cancer types was recently reviewed [199, 200].

## The safety of TLR agonist administration

Clinical trials revealed that the TLR agonists were generally well tolerated. However, side effects raised from treatments still affect the neurological function and life quality of patients. The most common adverse effects of Poly I:C administration include temporary injection site discomfort, grade 1/2 flu-like symptoms (fever, myalgia, nausea, vomiting, fatigue, and headache) [140, 143], which usually disappear within 1–2 week. Occasional Grade 3 fever was observed (NCT03392545) and was limited to 48 h after treatment. Grade 1 leukopenia was reported in some patients, and no instances of autoimmunity were encountered [140]. Poly ICLC combined with radiation and TMZ in adults with GBM (NCT00262730) showed that grade 3/4 toxicities include neutropenia, leukopenia, thrombocytopenia, and rash, which were considered common treatment-related toxicity. In children, the grade 1/2 adverse events caused by Poly ICLC and GAA vaccination include gastrointestinal toxicity, anemia, lymphopenia, and allergic reaction, while grade 3/4 toxicity was also reported such as urticaria, non-hematological toxicity, and hematologic toxicity [144, 145]. Mild elevation of liver transaminase after Poly ICLC administration was detected both in children and in adults [143]. No severe toxicity or agonist-related death was reported.

The toxicity associated with HSSP-96 administration was also shown to be minimal. Grade 3 fatigue, low fever, and cutaneous pruritus were the most common adverse events, grade 3 hemiplegia happened occasionally, while no grade 3/4 vaccine-related adverse events were noted [157, 158]. In recurrent GBM, HSSP-96 gave rise to grade 3–4 adverse event and subdural hematoma associated with surgical resection [158]. No death event attributed to the vaccine was reported.

Unlike other agonists which were usually applied through skin absorption or muscular injection, CpG-ODN can be administrated subcutaneously, intrathecally, or intracranially [173]. Application of CpG-ODN alone revealed grade 2 common adverse effects such as lymphopenia, anemia, and neutropenia, local erythema at injection sites, fever, and seizure [173]. CpG-28 was well tolerated, transient neurological worsening or fatigue was the most significant toxicity observed, while increased steroid levels and relatively severe arachnoiditis happened occasionally but was well tolerated [172]. Although most clinical trials reported reasonably tolerated toxicity, one needs to keep in mind that most studies were limited by the number of enrolled patients (mostly under 100 or much less), and larger randomized clinical studies need to be performed to overcome the limitation of small sample size and to fully evaluate the efficacy of therapy before one could confidently declare.

## Other TLR targets for glioma treatment

Alongside TLR-targeting agonists and immune checkpoints, the roles of other molecules have been investigated in TLR-mediated tumorigenesis, providing a wide spectrum of possible therapeutic options (Table 5). TLR-targeting factors effectively suppress glioma growth in vitro and/or in vivo studies include zoledronic acid [201], annexin A2 [202], ortho-vanillin [75], cannabinoids [114], transcription factor interferon regulatory factor 3 [203], paeoniflorin [204], prosaposin suppression [205], and chloroquine [126]. However, further preclinical studies are required to clarify the therapeutic effects of these agents.

**Table 5:** Other potential targets for TLR-based antitumor GBM treatment

Potential antitumor agent	Definition	TLR target	In vitro cell line(s)	In vivo model(s)	Effects and mechanisms	Ref
Zoledronic acid	Bisphosphonate that exhibits anticancer activity	TLR2	U87	n/a	Induced GBM cell apoptosis through overexpression of TLR2, interferon regulatory factor 5, and endoplasmic reticulum-nuclei-1	[201]
Ortho vanillin	An isomer of food supplement vanillin	TLR2	GL261 and primary microglial cells derived from C57Bl/6 mice	n/a	Suppressed glioma-induced TLR2 signaling and cell proliferation in GAM through suppression of MMPs, IL-6, and iNOS	[75]
Annexin A2	Phospholipid-binding proteins that regulate membrane organization	TLR2	GL261	Murine GL261 implanted C57BL/6 mouse model	Enhanced antigen-specific T-cell responses via TLR2 activation. Fusing Annexin A2 with TLR agonist may induce synergetic antitumor immunity	[202]
Cannabinoids	Compounds that function in inflammation and tumor progression	TLR2	U87	n/a	Inhibited PGN-induced TLR2-mediated NF-κB activation and tumor cell growth via CB1 cannabinoid receptor	[114]
HMGB1	Proinflammatory mediator released from dying tumor cells	TLR2	GL26, GL261	Murine GL261 implanted C57BL/6 mouse	Mediated TLR2-dependent glioma tumor regression associated with long-term survival in respond to Fms-like tyrosine kinase 3 ligand and thymidine kinase treatment	[84]
		TLR4	A72, U87	n/a	Contributed to immune evasion via antigen HLA-G regulation through interaction with TLR4	[63]
p62	Signaling adaptor sequestosome-1 that shuttles ubiquitinated targets	TLR4	T98G, primary GBM cells	Primary GBM G141119 glioma xenografts	Crucial for HMGB1-stimulated epithelial–mesenchymal transition and GBM cell invasion via TLR4-p38-Nrf2 activation	[162]
HSP90α	Extracellular heat shock protein	TLR4	U87	n/a	Favored TLR4-mediated GBM cell migration	[155]
Paeoniflorin	A polyphenol that exhibits anticancer activation	TLR4	U87, U251, U87-luciferase	Human U87-implanted BALB/c nude mouse	Inhibited GBM growth through TLR4/Triad3A-axis-mediated TLR4 degradation	[204]
Interferon regulatory factor 3	A transcription factor that is critical to induce antiviral immune responses	TLR4	U251, U87, SNB19	n/a	Suppressed glioma progression and regulates the expression of glioma cytokine, chemokine, and miR155 via TLR4-mediated signaling	[203]
Chloroquine	Inhibitor of endosomal maturation	TLR9	D54MG	n/a	Inhibited CpG or non-CpG-ODN-induced tumor cell invasion through TLR signaling	[126]

n/a indicates information that is not available

Lastly, it is noteworthy that some microRNAs (miRNA) have significantly elevated expression in glioma tissues and cell lines [206] and are closely associated with TLR-regulated immune responses, suggesting that these miRNAs could be potential targets for modern immunotherapy. For example, long noncoding RNA UBE2R2-AS1 targeted *TLR4* mRNA by binding to miR-877-3p, resulting in suppression of glioma cell growth, migration, and invasiveness [207]. A subset of let-7 miRNA that functions as TLR7 ligands was shown to induce microglial release of inflammatory cytokines and antigen presentation to suppress tumor cell migration and glioma growth [208]. Micro-RNA miR-21 limited PDCD4 expression via TLR4-signaling in response to LPS by inhibition of NF-κB and elevated IL-10 production [209]. LPS and miR-34a have been shown to induce the expression of miR-132, miR-155, and miR146a that downregulated TLR4 signaling and suppressed tumor invasion and migration [210, 211]. However, the suppressive roles of these miRNAs in glioma require further clarification. Recent review on TLR-targeted miRNA development is available [212], and a manually curated database NoncoRNA (http://www.ncdtcdb.cn:8080/NoncoRNA/) is accessible to search for potential therapeutic RNA targets in the treatment of human cancers including glioma [213].

## Challenges and future directions of TLR-based immunotherapy

### Challenges

Although TLR agonists have shown some promising results in preclinical and clinical studies, challenges remain to fully unlock the potential of TLR-based immunotherapy in glioma treatment. Up to date, TLR2, TLR3, TLR4, TLR7, and TLR9 have been intensely studied in glioma, while less or missing information is available regarding the mechanisms of TLR1/TLR6 (dimerizes with TLR2), TLR5, TLR8 (dimerizes with TLR7), and particularly TLR10. TLRs may present anti- or protumor activity, depending on the site of expressions. TLR overexpression on immune cells generally enhances antitumor effects, whereas high expression on GSCs and tumor cells is usually pro-tumorigenic [62]. Apart from classical ligands, TLRs can also be activated in vivo by various factors released from inflammation, such as hypoxia or necrosis, which are common pathologic events associated with the presence of dying glioma cells [108, 109, 128]. This leads to a complex situation where one molecule may interact with and activate the signaling of multiple TLRs [154], suggesting that extra care should be taken into agonist selection for antitumor effects and result analysis. TLR expression may also vary greatly between individuals, leading to differential benefits for patients receiving TLR-based treatment.

In addition, the tumor implantation site (subcutaneous or intracranial) in in vivo experiments may lead to substantial differences in the survival times of animal models, in which less robust antitumoral effects were usually observed against intracranial tumors due to the immune environment of the CNS [92]. In classical glioma models, the missing genetic markers, surgical process, and anatomical features do not adequately represent the complexity of human glioma, explaining the inconsistent results obtained in animal models and in human trials. Preclinical manipulations with representative tumor heterogeneity are required to provide more accurate guidance to clinical translation, and the experimental results must be interpreted with caution. Larger randomized clinical studies are also required to overcome the limitation of small sample size, whose results may be biased by confounding factors such as pseudo-progression or hyper-progression, and to evaluate fully the efficacy of TLR agonists.

### Future directions

The presence of blood–brain barrier is one of the key factors hampering effective delivery of chemotherapeutic drugs inside glioma. Recent in vitro and in vivo studies have focused on delivery or co-delivery of TLR agonists using nanoparticles, such as carbon nanotubes [179], nanodisc [181], and schizophyllan [180], which not only enhanced drug transport across the blood–brain barrier to target TLRs, but also elevated immune response.

Clinically, there is limited intratumoral infiltration of adaptive immune cells in glioma, presenting one of the major challenges in immunotherapy. The application of TLR agonists could proficiently work as vaccine adjuvant with broad innate and adaptive immune-enhancing effects via activation of APCs and effector T-cells [72, 76, 81]. Research efforts have been made to combine TLR agonists with vaccines of various types against the highly immunosuppressive glioma microenvironment and showed some promising clinical results [140–142]. Recently, Hilf and co-workers presented a more personalized vaccination based on individual patient’s transcriptome/immunopeptidome data to generate tumor-specific antigens together with TLR agonist as an immunomodulator to treat malignant GBM. Great immunogenicity and a much beneficial result was obtained [37], suggesting a promising future of using TLR agonist and personalized vaccination to treat HGGs.

Targeting the immune checkpoint PD-1/PD-L1 axis has been the center of spotlight in cancer treatment for their ability to protect against autoimmunity and immune escape. TLR signaling was discovered to enhance the expression of PD-1 and PD-L1 on glioma cells [183], indicating that TLR-based therapies combined with checkpoint inhibitor can be beneficial for treating against PD-1/PD-L1-expressing tumor cells. Activation of TLR3, TLR4, and TLR9 has been reported to reinforce the antitumor response to anti-PD-1/PD-L1 and exhibit immune checkpoint delayed resistance in glioma cells or animal models [187, 188, 190]. However, insufficient clinical information is available to make any conclusive statement regarding the efficiency and safety use of TLR-based checkpoint inhibition immunotherapy in human glioma patients. Future directions of TLR-based glioma treatment may also include the understanding of precise molecular mechanisms of how glioma cells have cross talk and influence TME function, and the development of therapeutics targeting glioma biomarker to better enhance the antitumor immune responses of TLR-based immunotherapy.

## Conclusion

Glioma, especially HGG, is presented as a challenging tumor type due to its high invasiveness, short OS, recurrence, and resistance to standard therapies. TLR signaling and their broad spectrum of interactions provide attractive approaches for glioma immunotherapy. Applications of TLR agonists to suppress pro-tumorigenic signaling and undesired GAM accumulation, to modulate TLR-expressing GSCs and immune cells for the creation of antitumor microenvironment, to induce PD-1/PD-L1 expression, all showed how many processes could be directly or indirectly modulated by targeting TLR signaling. Recent clinical studies on TLR-based immunotherapy have presented effective responses in some glioma patients, either used as stand-alone treatment, or combined with radiation therapy, chemotherapy, immune vaccination, or checkpoint inhibitor against the highly immunosuppressive glioma microenvironment. Although positive results have been observed, further clinical observations are required to overcome current deficiencies and create the best therapeutic strategies that can bring new hope for glioma patients. We anticipate that TLR-based treatments in the future will provide promising results for successful treatment of glioma.

## Data Availability

Not applicable.

## Abbreviations

APC: Antigen-presenting cell
CAR: Chimeric antigen receptor
CNS: Central nervous system
CpG-ODN: Cytosine–phosphate–guanosine oligodeoxynucleotide
DAMP: Danger-associated molecular pattern
DC: Dendritic cell
EAE: Experimental autoimmune encephalomyelitis
EGF: Epidermal growth factor
EGFR: Epidermal growth factor receptor
Erk1: Extracellular signal-regulated kinase
FGFR1: Fibroblast growth factor receptor 1
G100: Glucopyranosyl lipid A
GAA: Glioma-associated antigen
GAM: Glioma-associated microglia
GBM: Glioblastoma
GSC: Glioma stem cell
HGG: High-grade glioma
HMGB: High-mobility group box protein
HSP: Heat shock protein
IDH: Isocitrate dehydrogenase
IFN: Interferon
IFNAR: Interferon-α/β receptor
IGF: Insulin-like growth factor
IKK: IkappaB kinase
IL: Interleukin
iNOS: Inducible nitric oxide synthase
IRAK: IL-1R-associated kinase
IRF: Interferon regulatory factor
JNK: C-Jun kinase
LGG: Low-grade glioma
LPS: Lipopolysaccharide
LRR: Leucine-rich repeats
MAPK: Mitogen-activated kinase
mDC: Myeloid dendritic cell
MDSC: Myeloid-derived suppressor cell
MGMT: O-6-methyguanine-DNA methyltransferase
MHC I: Major histocompatibility complex class I
MMP: Matrix metalloproteinase
MS: Multiple sclerosis
MT1-MMP: Membrane-bound metalloproteinase
MyD88: Myeloid differentiation factor 88
NEMO: NF-κB essential modulator
OS: Overall survival
PAMP: Pathogen-associated molecular pattern
pDC: Plasmacytoid dendritic cell
PD-1: Programmed cell death protein 1
PD-L1: Programmed cell death ligand 1
PFS: Progression-free survival
PGN: Peptidoglycan
Poly(IC): Polyinosinic–polycytidylic acid
Poly ICLC: Polyinosinic–polycytidylic acid, and poly-L-lysine
PTEN: Phosphatase and tensin homolog on chromosome 10
RBBP5: Retinoblastoma-binding protein 5
RIP: Receptor-interacting serine/threonine protein
STAT3: Signal transducer and activator of transcription 3
TAB: TAK1-binding protein
TBK: TANK-binding kinase
TAK: Transforming growth factor β-activated kinase
TERT: Telomerase reverse transcriptase
Th: T-helper cell
TIR: Toll/interlekin-1 receptor
TLR: Toll-like receptor
TME: Tumor microenvironment
TMZ: Temozolomide
TNF: Tumor necrosis factor
TRAF: Tumor necrosis factor receptor-associated factor
Treg: Regulatory T-cell
TRIF: TIR-domain-containing adaptor-inducing interferon-β
VEGF: Vascular endothelial growth factor
WHO: World Health Organization
